# Effectiveness and feasibility of a theory-informed intervention to improve Mediterranean diet adherence, physical activity and cognition in older adults at risk of dementia: the MedEx-UK randomised controlled trial

**DOI:** 10.1186/s12916-024-03815-z

**Published:** 2024-12-23

**Authors:** A. Jennings, O. M. Shannon, R. Gillings, V. Lee, R. Elsworthy, R. Bundy, G. Rao, S. Hanson, W. Hardeman, S-M. Paddick, M. Siervo, S. Aldred, J. C. Mathers, M. Hornberger, A. M. Minihane

**Affiliations:** 1 Norwich Medical School, University of East Anglia, Norwich, UK; 2 School of Biological Sciences, The Co-Centre for Sustainable Food Systems and The Institute for Global Food Security, Queens University Belfast, Belfast, UK; 3 Behavioural and Implementation Science Group, School of Health Sciences, University of East Anglia, Norwich, UK; 4 Norwich Institute of Healthy Ageing, University of East Anglia, Norwich, UK; 5 Human Nutrition & Exercise Research Centre, Population Health Sciences Institute, Newcastle University, Newcastle Upon Tyne, UK; 6 School of Sport, Exercise and Rehabilitation Sciences, University of Birmingham, Birmingham, UK; 7 The George Institute for Global Health, Barangaroo, NSW Australia; 8 Faculty of Medicine, University of New South Wales, Kensington, NSW Australia; 9 Translational and Clinical Medicine, Newcastle University, Campus for Ageing and Vitality, Westgate Road, Newcastle Upon Tyne, UK; 10 Gateshead Health NHS Foundation Trust, Bensham Hospital, Saltwell Road, Gateshead, UK; 11 Curtin Dementia Centre of Excellence, enAble Institute, Curtin University, Perth, Australia

**Keywords:** Mediterranean diet, Physical activity, Behaviour change, RCT, Dementia

## Abstract

**Background:**

Despite an urgent need for multi-domain lifestyle interventions to reduce dementia risk, there is a lack of interventions which are informed by theory- and evidence-based behaviour change strategies, and no interventions in this domain have investigated the feasibility or effectiveness of behaviour change maintenance. We tested the feasibility, acceptability and cognitive effects of a personalised theory-based 24-week intervention to improve Mediterranean diet (MD) adherence alone, or in combination with physical activity (PA), in older-adults at risk of dementia, defined using a cardiovascular risk score.

**Methods:**

Participants (*n* = 104, 74% female, 57–76 years) were randomised to three parallel intervention arms: (1) control, (2) MD, or (3) MD + PA for 24 weeks and invited to an optional 24-week follow-up period with no active intervention. Behaviour change was supported using personalised targets, a web-based intervention, group sessions and food provision. The primary outcome was behaviour change (MD adherence and PA levels), and the secondary outcomes included feasibility and acceptability, cognitive function, cardiometabolic health (BMI and 24-h ambulatory blood pressure) and process measures.

**Results:**

The intervention was feasible and acceptable with the intended number of participants completing the study. Participant engagement with group sessions and food provision components was high. There was improved MD adherence in the two MD groups compared with control at 24 weeks (3.7 points on a 14-point scale (95% CI 2.9, 4.5) and 48 weeks (2.7 points (95% CI 1.6, 3.7)). The intervention did not significantly change objectively measured PA. Improvements in general cognition (0.22 (95% CI 0.05, 0.35), memory (0.31 (95% CI 0.10, 0.51) and select cardiovascular outcomes captured as underpinning physiological mechanisms were observed in the MD groups at 24 weeks.

**Conclusions:**

The intervention was successful in initiating and maintaining dietary behaviour change for up to 12 months which resulted in cognitive benefits. It provides a framework for future complex behaviour change interventions with a range of health and well-being endpoints.

**Trial registration:**

ClinicalTrials.gov NCT03673722.

**Supplementary Information:**

The online version contains supplementary material available at 10.1186/s12916-024-03815-z.

## Background

Dementia is a major public health concern with a substantial social and economic cost [1]. Given the considerable and rising prevalence of this condition, the identification of feasible, acceptable, and effective dementia prevention strategies is a major research priority [2]. Whilst promotion of healthy lifestyles across the life course is essential, given the ageing population, it is especially important that middle-aged and older adults are supported to modify behavioural risk factors, in particular, to mitigate dementia risk [2]. Mid-to-later life is acknowledged as an important window for dementia prevention, with many dementia risk factors occurring during this period [3]. Dietary/PA changes during midlife through to early later life could be advantageous (compared with later intervention) by allowing healthy behaviours to be maintained for a longer period [4].

Recent large-scale, multi-domain interventions comprising dietary and physical activity (PA) changes, alongside other intervention components (e.g. cognitive training [5, 6] and cardiovascular risk management [5, 7]), have been shown to reduce dementia risk in older at-risk participants [5–7]. This includes benefits in the entire cohort in the FINGER study [5] and in specific population sub-groups in the MAPT (participants with an elevated CAIDE score) [6] and Pre-DIVA (participants with baseline untreated hypertension) [7] trials. Meanwhile, isolated intervention with a Mediterranean diet (MD) has been shown to improve cognitive function in older adults [8, 9]. In addition, our previous prospective cohort research indicated higher MD adherence was associated with up to five fewer years of cognitive ageing [10] and lower dementia risk [11].

It is hypothesised that simultaneously improving both dietary behaviours and PA levels could have additive and synergistic effects on brain health through overlapping physiological processes and activation of common mechanistic pathways [12, 13]. These mechanisms include systemic benefits which may indirectly aid brain health such as improvements in cardiometabolic health (e.g. lower blood pressure and greater endothelial function) [14–16], lower levels of inflammation and oxidative stress [17, 18] and modulation of the composition and associated metabolome of the gut microbiota [19, 20]. In addition, diet and PA could directly impact the brain by improving blood brain barrier function, enhancing cerebral blood flow, reducing small vessel disease, promoting the induction of brain derived neurotrophic factor (a neuroplasticity biomarkers) and increasing β-amyloid clearance [18, 21–25].

To our knowledge, only one previous intervention in Australia has examined the impact of a combined intervention to increase MD adherence and PA on neurocognitive function, with none conducted in the UK [26]. In addition, there are also a lack of combined MD and PA interventions which are informed by theory- and evidence-based behaviour change strategies, and no interventions in this domain have investigated the feasibility or effectiveness of behaviour change maintenance. Various barriers make adoption of a MD in a non-Mediterranean setting challenging, including cultural identity, perceived time available for cooking, cooking skills, changes to traditional dining patterns, the cooler climate and the cost, availability and acceptability of MD components, which necessitates careful intervention development with the MD (alone or alongside increased PA) [27, 28].

In the current manuscript, we report the primary and secondary outcomes of the MedEx-UK study, a 24-week multi-domain, theory-based intervention to improve MD adherence alone, or in combination with PA, in older adults at risk of dementia. Following the 24-week intervention, we invited all participants to a further 24-week follow-up period with limited intervention (continued access to a web-based module) to investigate behaviour change maintenance in response to the MedEx-UK intervention. The primary outcome was behaviour change (MD adherence and PA levels), and the secondary outcomes included feasibility and acceptability of the intervention, cognitive function, cardiometabolic health (BMI and 24-h ambulatory blood pressure) and process measures such as theory-based mediators of behaviour change. Data are presented for outcomes at both 24- and 48-week follow-up.

## Methods

### Study design

The study was pre-registered with ClinicalTrials.gov (NCT03673722), and the details of the protocol have been published [29]. The reporting of this study follows the CONSORT for reporting randomised trials guidelines. Briefly, participants from three UK centres (Norwich, Newcastle, and Birmingham) were randomised to a personalised, multi-domain intervention into one of three parallel intervention arms: (1) control, (2) MD, and (3) MD + PA. The main 24-week intervention took place between March 2019 and September 2020, and the 24- to 48-week trial add-on behaviour maintenance phase was completed by March 2021.

The sample size calculation for the study, based on dietary change of three-points on the Mediterranean Diet Adherence Screener (MEDAS), indicated 90 participants (30 participants in each arm) would be required to complete the study (90% power and 5% error), which was increased to 108 participants to account for a 20% drop-out rate [29]. With this sample size, the smallest detectable change in MEDAS score was 1.23 points. For physical activity, we estimated that this sample size would allow us to detect a change in moderate activity per week with a confidence interval of 45 min [30] suggesting we were powered to detect a change in PA from under 60 min to over 150 min per week.

### Participants

Individuals aged 55 to 74 years were recruited to take part in the intervention through primary care, in collaboration with the local Clinical Research Networks at each study site, and via direct-to-public advertisements. Full details of the study inclusion/exclusion criteria are presented in Additional file 1: Methods S1. As cardiovascular risk scores have established associations with *dementia* and cognitive impairment [31], we defined participants at risk of dementia as having a cardiovascular risk score (QRISK2) ≥ 10%, which indicates a ≥ 10% risk of having a cardiovascular event in the next 10 years [32]. The QRISK2 score, routinely used in UK primary care, accounts for a number of risk factors for cardiovascular disease, including age, gender, ethnicity, hypertension, cholesterol, BMI, smoking, alcohol intake and presence of medical conditions such as diabetes, rheumatoid arthritis, and chronic kidney disease. As the intervention was focussed on primary prevention, and mid-life to younger-old age (< 75 years) rather than older age cardiometabolic health is an important modifiable risk factor for dementia [3], participants aged 55–74 years were recruited. Participants were also required to have (1) normal cognitive function as determined by a Montreal Cognitive Assessment score ≥ 23 [33]; (2) no mild cognitive impairment, dementia or other severe neuropsychological complaints (as detailed in Additional file 1: Methods S1); (3) a baseline MEDAS score < 9 according to a modified version of MEDAS [34]; and (4) < 90 min self-reported moderate-intensity PA each week. Eligibility to participate was determined through online, telephone and in-person screening sessions. For the participants recruited through primary care, we were able to use existing health records to determine cardiovascular risk and selected other health endpoints which were exclusion criteria. Information on age, sex, deprivation (from postcode) and race (White, Indian, Pakistani, Bangladeshi, Other Asian, Black Caribbean, Black African, Chinese or other ethnic groups) were self-reported.

### Randomisation

Individuals who were deemed eligible to participate in MedEx-UK were allocated randomly to one of the three study intervention arms, with minimisation for MEDAS score (low = 0–4; high = 5–8) and sex whilst stratified by site, to ensure treatment arms were balanced for these parameters, using a computerised random number function in Microsoft Excel. Randomisation and allocation were completed by researchers who were not blinded to group assignment.

### Intervention phase

The first 24 weeks of the study comprised an intensive intervention period, during which participants in the MD and MD + PA arms were encouraged to change their behaviour via a combination of personalised goals, a web-based intervention, group sessions with facilitators trained in behaviour change techniques and supermarket vouchers or food delivery to support behaviour change. Subsequently, participants were invited to take part in a behavioural maintenance phase (weeks 24–48), during which they had continued access to the web-based intervention only.

The intervention targets were to improve MEDAS scores by at least three points and increase levels of activity to 150 min of moderate, or 75 min of vigorous, activity per week. Participants were encouraged to select their own goals to meet these targets, which were introduced in a gradual process. As part of the website intervention, participants were asked to self-assess their consumption of the Mediterranean diet and their PA levels. They subsequently received personalised feedback from the trained facilitators during group sessions and the web-based platform described below.

The web-based intervention was administered via an interactive, modular platform called LEAP^2^, as described elsewhere [29]. LEAP^2^ included the ‘Eating Well’ module, designed to help participants increase their MEDAS score by providing real-time access to their score and details of the goals they were meeting, and facilitating participants to choose their own goals based on individual food preferences. Full details of the MEDAS targets are presented in Additional File 1: Table S1. Due to the negative associations between alcohol consumption and brain health, participants were asked not to increase or change their alcohol intake but if they consumed alcohol to switch the type of alcohol they consumed to wine, preferably red wine.

The ‘Moving More’ module (accessible only by participants in the MD + PA arm) was designed to help participants increase their PA. The module included a questionnaire to allow participants to determine their current PA levels and receive an award based on the level achieved (bronze (≥ 100 min of moderate or 50 min of vigorous-intensity PA per week), silver (≥ 120 min of moderate or 60 min of vigorous-intensity PA per week) or gold (≥ 150 min of moderate or 75 min of vigorous-intensity PA per week). Participants were encouraged to set a goal of moderate and/or vigorous activity in minutes per week, and LEAP^2^ provided tailored PA suggestions based around participants preferences for cost, intensity and type (group or individual) of exercise and guided participants through overcoming key barriers associated with increasing PA levels.

In addition, LEAP^2^ included a diary feature to help participants plan meals and PA and links to the study dietary assessment tool (Intake24) and the food provision element of the MedEx-UK study. Participants were encouraged to visit LEAP^2^ regularly throughout the 24-week intervention period.

Participants in the MD and MD + PA arms were invited to attend four group sessions (at weeks 0, 2, 4 and 12) that were designed to complement the web-based intervention. The group sessions were 2 h for the MD group and 2.5 h for the MD + PA group and comprised ~ 6 participants and ~ 6 supportive others (i.e. a friend or relative to provide social support). The group sessions were designed to target key influences on behaviour change based on the Capability, Opportunity, Motivation and Behaviour (COM-B) Model [35] and incorporated evidence-based behaviour change techniques to encourage change and maintenance of any changes [35, 36].

Due to the COVID-19 pandemic, group sessions were conducted both in-person (prior to March 2020) and via videoconferencing software (after March 2020 during ‘lockdown’ periods). Participants were notified of their intervention group allocation at the start of their first group session and therefore were blinded to group allocation at baseline but not follow-up assessments. Researchers conducting measurements were not blinded because of practical impossibilities, including the fact that participants themselves were aware of group assignment. However, the two primary outcomes, namely eating behaviour and PA, were assessed by self-administered questionnaire and activity monitors respectively, with no researcher input.

Finally, participants in the MD and MD + PA groups were provided with £30 per week in vouchers for an online food retailer. Participants were encouraged to purchase foods that contributed to their MEDAS target score, but this was not monitored. In cases where online food delivery was not possible (e.g. due to delivery restrictions to rural areas), participants were provided with equivalent vouchers for a supermarket of their choice.

Participants in the control group received dietary and PA advice in accordance with the UK National Institute for Health and Care Excellence (NICE) guidelines for individuals with a moderately elevated QRISK2 score [37]. They also attended a 1-h group session at week 0, during which they were informed of their intervention group allocation and received a brief verbal presentation outlining the importance of a control group in research. Following completion of the 24-week intervention phase, the control group received £240 shopping vouchers (equivalent to £10 per week for participation) as remuneration.

### Behavioural maintenance phase sub-study

In an optional sub-study following the initial 24-week intervention period, consenting participants entered a behavioural maintenance phase during which they had continued access to the LEAP^2^ platform but no longer received group support sessions or food provision. The LEAP^2^ platform was modified to include content which aimed to support participants in maintaining healthy behaviour change achieved during the initial study intervention period; full details are provided in Additional File 1: Methods S2. This study maintenance phase was a trial add-on initiated after participants were recruited and consented to the main 24-week intervention.

### Outcomes

Baseline assessments were conducted between September 2019 and March 2020 in-person at a clinical testing facility during which eating and PA behaviours, cognitive function, cardiometabolic health (BMI and 24-h ambulatory blood pressure) and biological outcomes were measured (1). Due to the onset of the COVID-19 pandemic in March 2020, adaptions were made to the study protocol to minimise participant-researcher contact and to ensure compliance with social distancing restrictions. Data collection for the primary outcomes, dietary and physical activity behaviour change, were not changed from the protocol. For the 24- and 48-week assessments that took place between March 2020 and March 2021, only a sub-set of secondary measurements, including cognitive function, BMI and process evaluation, were obtained via remote (i.e. at-home) data collection. Specific adaptations have been highlighted for each measurement below. We were not able to collect data on 24-h ambulatory blood pressure (at 48 weeks), neuroimaging, vascular function or biological samples (including cholesterol for assessment of QRISK2) at 24 or 48 weeks, and therefore these data are not presented.

### Dietary assessment

Dietary intake, to determine level of adherence to the MD, was evaluated via two different approaches. Firstly, participants completed an online version of the 14-point MEDAS questionnaire [34], which was the primary dietary outcome measure in this study. Secondly, participants completed a series of 24-h recalls (on five non-consecutive days at baseline and at 24 and 48 weeks) via Intake24, a validated online dietary assessment tool [38]. These data were also used to calculate adherence to the 14-point MEDAS scale as detailed in Additional file 1: Table S1.

### Physical activity

PA levels were recorded for all participants throughout the entire intervention and behaviour maintenance periods via wrist worn activity monitors (Vivosmart 3, Garmin). The activity monitors were set to show the time and date only, to prevent participants receiving any activity-based feedback. Age, height and weight were entered when setting up the devices to improve accuracy. The devices recorded total step count, heart rate and PA energy expenditure. In addition, total activity levels in minutes of moderate intensity PA per week were calculated as follows: moderate minutes (defined as 40–59% heart rate reserve) + (vigorous minutes (≥ 60% heart rate reserve)*2) [39].

### Feasibility and acceptability

The feasibility of the intervention was assessed using recruitment and retention rates.

Intervention fidelity and participant engagement were evaluated via group session attendance (intervention phase) and self-reported usage of LEAP^2^ (intervention and behaviour maintenance phases) in the MD and MD + PA groups. Acceptability of the intervention was assessed at 24 and 48 weeks by a custom questionnaire using 5-point Likert-type scales, informed by the Theoretical Framework of Acceptability [40].

### Cognitive function

Cognitive function was determined using an extended version of the neuropsychological test battery (NTB) [29] measured at baseline and 24 and 48 weeks (Additional file 1: Methods S3).

Additionally, we included assessments of spatial navigation via the virtual reality Supermarket Trolley Task [41] and the Sea Hero Quest Test [42] and a further measure of executive function via the Hayling test. The duration of each cognitive assessment was approximately 90 min. Baseline assessments were conducted in-person at a clinical testing facility, and follow-up assessments at 24 and 48 weeks were conducted remotely via video conferencing software, to reduce in-person contact whilst COVID-19 social distancing measures were in effect. A researcher was present virtually during the testing, and paper-based cognitive tests were posted to participants before the session. It was not possible to collect data on the spatial navigation tasks during these remote sessions.

Scores from each test were converted to *Z* scores standardised on baseline grand mean and standard deviation. Response time variables on the Trail Making Test were reversed [*Z*-score * − 1], so for all cognitive tests, higher scores indicated better outcomes. Individual *Z* scores test scores were mean aggregated into summary scores for the following: Processing speed [Digit symbol substitution (total correct), Trail Making Test (A, seconds)], Executive Function [Controlled Oral Word Association Test (total), Categorical verbal fluency test (total), Trail Making Test (B-A, seconds), Wechsler Memory Digit Span (backwards, total)] and Memory [Visual paired (immediate and delayed totals), Verbal paired (immediate and delayed totals), Rey Auditory Verbal Learning Test (immediate and recall)]. A general cognition score was calculated as an average of the Processing Speed, Executive Function and Memory scores providing a weighted average across the three domains. Summary scores were only calculated for the participants who had completed all tests within each domain.

### BMI

At baseline, height and weight were measured after an overnight fast by a member of the research team using standard laboratory techniques and used to calculate body mass index (BMI). At 24 and 48 weeks, due to social distancing measures during the COVID-19 pandemic, participants were asked to measure their body weight at home using either their own electronic scales or those provided by the research team.

### 24-h ambulatory BP

Twenty-four-hour ambulatory blood pressure was measured at baseline and week 24 using portable devices (Mobil-O-Graph, Stolberg, Germany and Spacelab Healthcare, Washington, United States) which consisted of an inflatable cuff attached to a small monitoring system. The cuff was secured around the upper arm and readings were taken every 20 min during daytime (06:00 to 22:00) and every hour overnight (22:00 to 06:00) for an entire 24-h period.

### Process evaluation

The process evaluation was informed by UK Medical Research Council guidance for process evaluation [43]. Here, we present the quantitative measures related to mechanism of impact. The findings from interviews with group session facilitators and focus groups with participants, which focus additionally on contextual factors and implementation (e.g. fidelity), will be reported separately. Hypothesised mediators of behaviour change (intention, perceived control and self-reported use of behaviour change techniques) were assessed in all groups at baseline (intention and perceived control only), 24 and 48 weeks using 5-point Likert-type scales.

### Statistical analyses

Between-group differences in group-session attendance and use of the online-platform were examined between the MD + PA and MD groups using a 2-sample *t*-test or *χ*^2^ test for categorical data. The effect of the intervention on eating behaviour and other outcomes at 24 and 48 weeks were assessed using ANCOVA, with the 24-week value as the dependent variable and group as the independent variable. For the primary outcomes (change in MEDAS score and PA levels at 24 weeks), we repeated the analysis using intention to treat analysis with baseline values carried forward. For all analyses, we compared the difference in the mean of the control group with the mean of the two intervention groups (MD + PA and MD) (contrast 1) and the mean of the MD + PA with the mean of the MD group (contrast 2). Covariates included baseline value, study site and baseline BMI. The cognitive outcomes were additionally adjusted for age and years of education. We checked for effect modification by sex by including an interaction term for group*sex in the models of our key outcomes. Data are presented as the difference in mean values at 24 or 48 weeks for the two intervention groups (mean MD + PA and MD) minus the control group.

For eating behaviour change, we also included an interaction term for group*continuing to 48 weeks (y/n) in the model to examine if participants who continued to the maintenance phase were those with better outcomes at 24 weeks. We also calculated the percentage of participants who changed their diets sufficiently to meet the criteria for individual MEDAS components at 24 weeks. Finally, participants across all three groups (control, MD + PA, MD) were assigned to tertiles of 24-week change in MEDAS score and minutes of moderate activity and associations with cognitive and cardiometabolic outcomes at 24 weeks were examined. To account for multiple testing in these exploratory analyses, we calculated false discovery rate-adjusted *P* values using the Benjamin–Hochberg procedure.

All data are presented as unadjusted mean (SD) at individual timepoints, change (95% CI) or percentages where indicated. All analyses were performed using STATA (version 16; StataCorp LLC: College Station, TX).

### Role of the funding source

The funders of the study had no role in the study design, data collection, data analysis, data interpretation or writing of the report.

## Results

Of the *n* = 2776 participants who completed online screening, *n* = 239 met the criteria and attended in-person screening, and *n* = 104 (74% female, 57–76 years (mean 67.4 years (SD 4.6), 99% White, 15.2 years education (SD 3.1)) were recruited to the MedEx-UK study between 15 April 2019 and 10 January 2020. The main 24-week intervention was completed by *n* = 99 (5% drop out rate) of whom *n* = 76 (77%) consented and *n* = 69 (9% drop out rate of the 76 participants who consented to the 25–48-week maintenance phase of the study) completed the 24 to 48-week trial add-on behaviour maintenance phase (Additional file 1: Fig. S1 and Table 1). Complete data for the change in dietary and PA behaviours were available for *n* = 87 completers (88%) at 24 weeks and *n* = 52 completers (75%) at 48 weeks.

**Table 1:** Baseline characteristics of the MedEx-UK study participants, according to intervention group^a^

Characteristic	MD + PA (*n* = 35)	MD (*n* = 35)	Control (*n* = 34)
Sex, female	25 (71.4%)	27 (77.1%)	25 (73.5%)
Age, years	68.1 (5.1)	67.3 (4.3)	67.1 (4.4)
Race, White	35 (100%)	35 (100%)	33 (97%)
IMD, decile	5.9 (3.1)	5.2 (2.6)	6.3 (2.3)
Education, years	15.1 (2.8)	15.3 (2.7)	15.2 (3.7)
BMI, kg/m^2^	27.5 (4.2)	30.1 (5.0)	28.4 (3.5)
Current smoking, no	29 (87.9%)	30 (96.8%)	(96.4%)
MEDAS, score	6.8 (2.2)	5.9 (2.0)	6.8 (2.1)
Moderate activity, min/week	181 (154)	230 (205)	261 (308)
QRISK2, score	17.2 (5.8)	16.7 (4.9)	15.9 (4.7)
Type II diabetes, yes	1 (3.0%)	6 (19.4%)	1 (3.6%)
Blood pressure medication, yes	9 (27.3%)	13 (41.9%)	8 (28.6%)
General cognition score, *z*-score	− 0.07 (0.5)	0.12 (0.6)	− 0.01 (0.6)
Processing speed score, *z*-score	− 0.14 (0.8)	0.09 (0.9)	0.05 (0.9)
Executive function score, *z*-score	− 0.12 (0.6)	0.13 (0.8)	− 0.01 (0.6)
Memory score, *z*-score	0.05 (0.7)	0.05 (0.6)	− 0.08 (0.7)
24 h mean systolic BP, mm Hg	128 (12.5)	127 (14.3)	128 (11.1)
24 h mean diastolic BP, mm Hg	78.3 (10.3)	77.0 (9.6)	75.2 (9.8)
24 h mean pulse pressure, mm Hg	51.2 (9.3)	52.2 (10.9)	53.0 (7.5)
24 h systolic BP variability, mm Hg	10.8 (2.9)	10.6 (2.5)	10.1 (2.9)
24 h diastolic BP variability, mm Hg	11.0 (3.7)	11.0 (4.3)	10.8 (3.5)
24 h pulse pressure variability, mm Hg	16.3 (11.9)	13.9 (8.9)	14.1 (8.0)
Ambulatory Arterial Stiffness Index	0.61 (0.18)	0.58 (0.19)	0.51 (0.29)

^a^Values are mean (SD) or *n* = (%). Data was missing for BMI (*n* = 1 MD + PA), IMD (*n* = 1 MD + PA; *n* = 1 MD), moderate activity (*n* = 1 MD + PA; *n* = 2 control), current smoking, QRISK2, Type II diabetes and blood pressure medication (*n* = 4 MD + PA; *n* = 3 MD; *n* = 5 control), 24 hr blood pressure (*n*=4 MD+PA; *n*=3 MD; *n*=4 Control), 24 hr pulse pressure (*n*=4 MD+PA; *n*=5 MD; *n*=4 Control). *IMD* Index of Multiple Deprivation, *MD* Mediterranean diet, *MEDAS* Mediterranean Diet Adherence Screener, *PA* Physical activity, *QRISK2* Cardiovascular risk score

### Eating behaviour

**Fig. 1:**
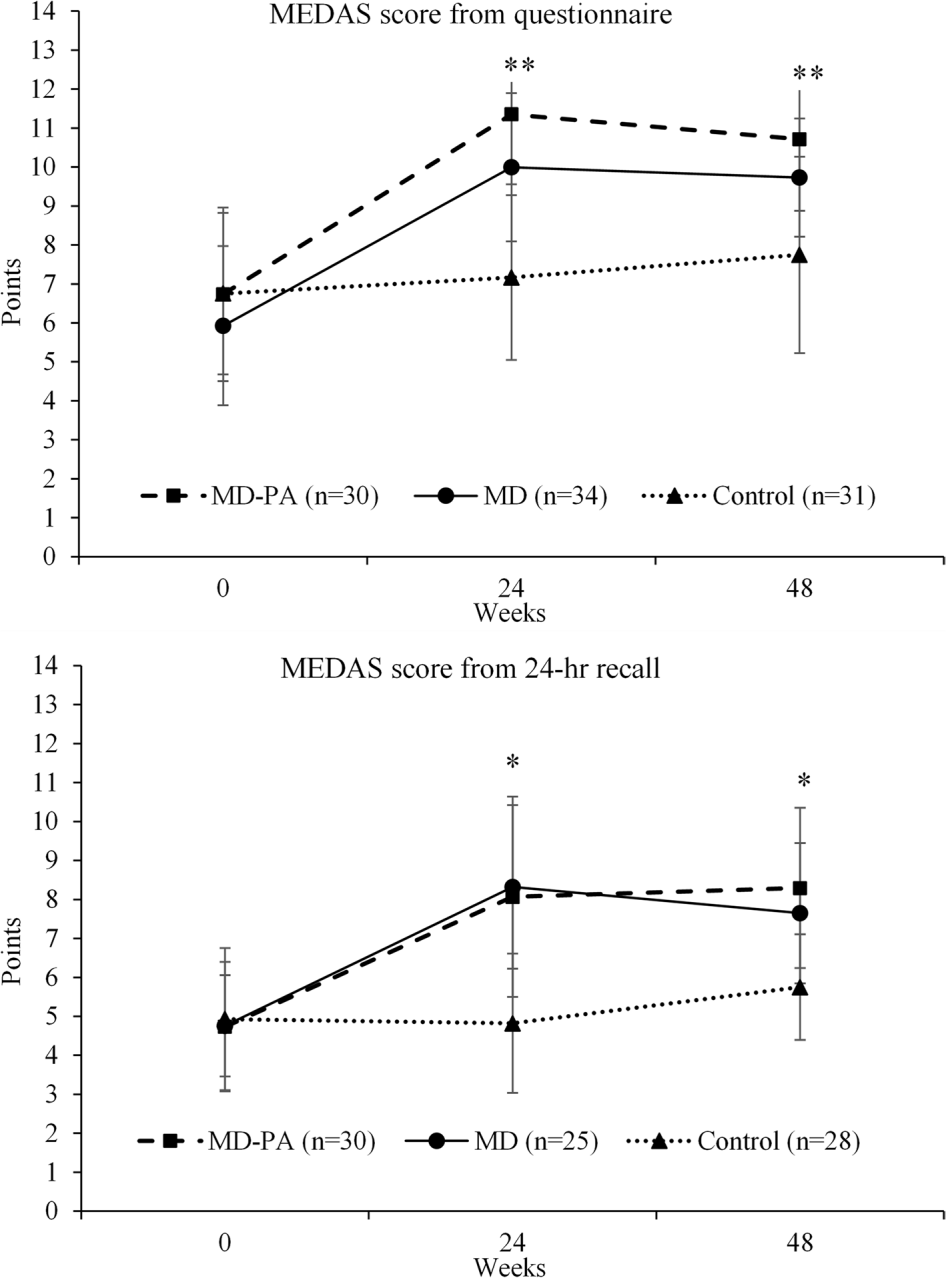
Mediterranean Diet Adherence Screener (MEDAS) score by intervention group at baseline and 24 and 48 weeks calculated by questionnaire and 24-h recall. Values represent unadjusted means (SD) from MEDAS questionnaire (**A**) and 24-h recall (**B**). *P*-value for group and contrast 1 (control v. (MD + MD + PA)) * < 0.01 or ** < 0.05 at relevant time point compared to baseline, calculated using ANCOVA (adjusted for baseline value, study site and baseline BMI). *P*-values for contrast 2 (MD v. MD + PA) were non-significant at all timepoints compared to baseline as were all contrasts comparing values at 48 to 24 weeks. Participant numbers at 48 weeks were *n* = 20 MD + PA, *n* = 22 MD, *n* = 21 control for questionnaire data and *n* = 17 MD + PA, *n* = 17 MD, *n* = 12 control for 24 = hr recall data. MD, Mediterranean diet; MEDAS, Mediterranean Diet Adherence Screener; PA, physical activity

After the 24-week intervention, there was improved MD adherence in the two MD groups compared with control when assessed using the MEDAS questionnaire (3.7 points (95% CI 2.9, 4.5) Fig. 1A and Additional File 1: Tables S2-3) and using 24-h recall (3.4 points (95% CI 2.4, 4.4) (Fig. 1B and Additional file 1: Tables S3-4). There was no evidence of a group by sex interaction (data not shown). Likewise, at 48 weeks, there was improved adherence in the two MD groups compared with control when assessed using the MEDAS questionnaire (2.7 points (95% CI 1.6, 3.7)) and using 24-h recall (2.6 points (95% CI 1.5, 3.8)) data (Fig. 1A, B). Participants in the MD group who participated in the maintenance phase had significantly higher MEDAS scores at 24 weeks compared to those who did not continue (between group difference of 1.5 points (95% CI 0.4, 2.8)), with no significant difference in the MD + PA group (between group difference of 0.9 points (95% CI − 0.4, 2.3)), although no significant interactions between group and continuing to maintenance phase were observed (data not shown).

**Fig. 2:**
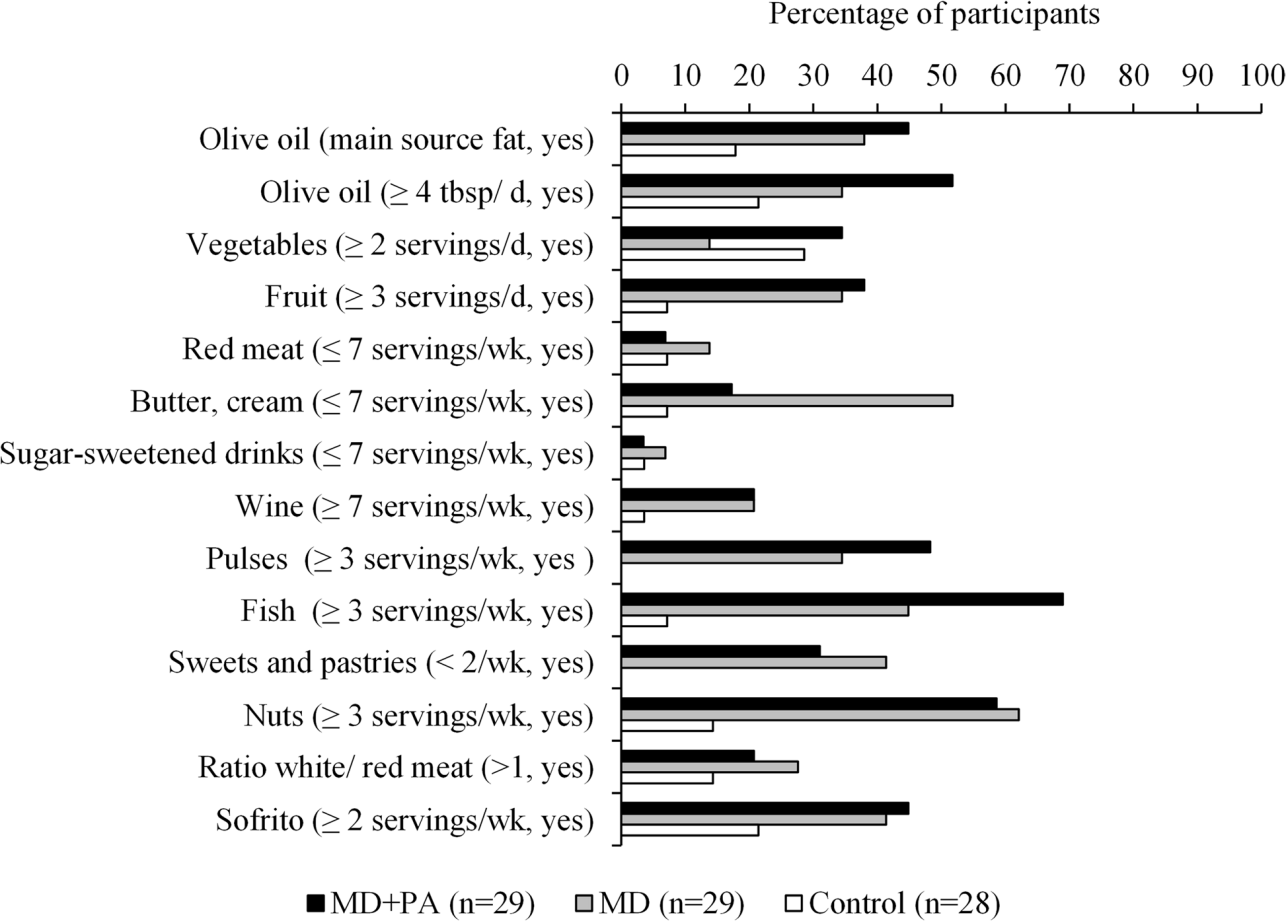
Proportion of participants adapting to meet the criteria for individual Mediterranean Diet Adherence Screener components at 24 weeks by intervention group in 86 MedEx-UK participants. Bars represent the percentage of participants who met the criteria at 24 weeks but not at baseline according to the questionnaire data. Only participants with complete data for all components were included (*n* = 86). Missing bars indicate the percentage of participants was zero

Scores for the individual components of the MEDAS improved in the MD diet groups compared with control over the 24-week intervention with the exception of the vegetable and sugar-sweetened drink components (when assessed by questionnaire) and the sugar-sweetened drink and butter and cream components (when assessed by 24-h recall) (Additional file 1: Tables S2-3). According to the MEDAS questionnaire data, low red meat and sugar-sweetened beverage intake was the recommendation met by the highest proportion of participants at baseline (Additional file 1: Fig. S2), and increasing nut and fish intake was the component that most participants in the MD groups changed, with 60% and 57%, respectively, adapting their diet sufficiently to meet the recommendations over the 24-week intervention (Fig. 2). Using 24-h recall data, the ratio of white to red meat and sofrito were the components that were most likely to be adapted (Additional file 1: Fig. S3).

### Physical activity

Total number of steps, energy expenditure and minutes of moderate activity increased in the MD + PA group and decreased in the MD and control groups after the 24-week intervention, but no significant between-group differences were observed (Table 2 and Additional file 1: Table S4). Likewise, at 48 weeks, no significant between-group differences were observed in PA (Table 2). There was no evidence of a group by sex interaction for minutes of moderate activity at 24 weeks, and there was no difference in minutes of moderate activity at 24 weeks between the participants in the MD + PA group who did, or did not, continue to 48 weeks (data not shown).

**Table 2:** Physical activity and cardiometabolic outcomes at baseline and 24 and 48 weeks by intervention group in 99 MedEx-UK participants

	**MD + PA**			**MD**			**Control**			**P1**^**1**^	**P2**^**2**^	**P3**^**3**^
	***n* = **	**Baseline**	**24 weeks**	***n* = **	**Baseline**	**24 weeks**	***n* = **	**Baseline**	**24 weeks**			
Total steps, d	28	5743 (2100)	6348 (3343)	31	6137 (2364)	5760 (2501)	31	6282 (2562)	5913 (2680)	0.13	0.23	0.31
Energy expenditure, kcal/week	28	281 (171)	327 (304)	31	322 (189)	277 (154)	30	303 (189)	239 (131)	0.06	0.12	0.30
Moderate activity, min/week	28	191 (163)	262 (183)	31	233 (214)	228 (195)	31	256 (311)	213 (210)	0.10	0.14	0.49
BMI, kg/m^2^	33	27.6 (4.2)	27.1 (4.4)	34	30.3 (5.0)	30.3 (5.2)	32	28.4 (3.6)	28.3 (3.4)	0.20	0.67	0.06
24 h mean SBP, mm Hg	13	128 (14.0)	125 (14.4)	13	128 (14.2)	130 (15.5)	11	131 (9.8)	128 (10.6)	0.57	0.58	0.04
24 h mean DBP, mm Hg	13	75.7 (10.0)	75.4 (10.1)	13	80.2 (10.7)	75.8 (6.0)	11	76.4 (7.5)	75.1 (4.8)	0.89	0.67	0.66
24 h mean PP, mm Hg	13	52.4 (7.3)	51.4 (7.2)	13	51.4 (11.0)	53.7 (12.1)	11	54.7 (7.8)	53.1 (8.2)	0.87	0.37	0.08
24 h SBP CV, mm Hg	13	10.5 (2.7)	9.5 (1.8)	13	9.6 (1.5)	9.6 (1.8)	11	9.4 (1.9)	9.6 (1.8)	0.32	0.49	0.41
24 h DBP CV, mm Hg	13	10.2 (3.2)	9.8 (3.6)	13	9.5 (2.6)	10.4 (2.7)	11	10.6 (2.9)	10.6 (2.6)	0.32	0.85	0.10
24 h PP CV, mm Hg	13	14.0 (9.9)	11.4 (6.9)	13	15.6 (8.3)	15.7 (9.1)	11	14.1 (7.4)	15.9 (8.4)	< 0.01	0.02	0.02
AASI	13	0.58 (0.19)	0.50 (0.10)	13	0.62 (0.17)	0.64 (0.16)	11	0.60 (0.20)	0.64 (0.14)	0.01	0.13	< 0.01
			**48 weeks**			**48 weeks**			**48 weeks**			
Total steps, d	17	5497 (2187)	5470 (2647)	21	6264 (2055)	5683 (2499)	18	5895 (2284)	5586 (2822)	0.85	0.81	0.96
Energy expenditure, kcal/week	17	228 (97)	242 (134)	21	310 (165)	289 (201)	16	301 (166)	248 (195)	0.56	0.66	0.67
Moderate activity, min/week	17	204 (167)	246 (247)	21	216 (209)	314 (228)	18	273 (316)	288 (374)	0.59	0.37	0.61
BMI, kg/m^2^	24	27.0 (3.2)	26.3 (3.2)	23	30.3 (4.7)	29.9 (4.9)	21	27.8 (3.2)	28.0 (3.2)	< 0.01	0.02	0.31

Values are unadjusted means (SD). Moderate activity minutes have been derived from light, moderate and vigorous intensity minutes normalised to moderate intensity

*AASI* Ambulatory stiffness index, *CV* Variability, *DBP* Diastolic blood pressure, *MD* Mediterranean diet, *PA* Physical activity, *PP* Pulse pressure, *SBP* Systolic blood pressure

^1^P1 = *P*-value for group using ANCOVA (adjusted for baseline value, study site and baseline BMI)

^2^P2 = *P*-value for contrast 1: control v. (MD + MDPA)

^3^P3 = *P*-value for contrast 2: MD v. MDPA

### Feasibility and acceptability

Engagement with the group sessions was high, with participants attending 3.5 (SD 0.9) of the four group sessions in both the MD + PA and MD groups (Additional file 1: Table S5). Most participants (84%) reported accessing the online platform once per month or less, with the average session length 15 to 30 min. Uptake of food delivery or supermarket vouchers was 100% each month. Ninety-five percent of participants reported the intervention to be acceptable, with no significant difference between the MD and MD + PA intervention groups (Additional file 1: Fig. S4). Overall, participants rated the acceptability of the online platform lower than other intervention components (score 3.2 out of possible 5, compared to 4.0 for group sessions and 3.8 food delivery) (Additional file 1: Table S6). Participants rated their understanding of how the intervention aimed to facilitate behaviour change highly and reported a good fit with their beliefs about behaviour change (both average scores 4.3 out of possible five, Additional file 1: Table S6).

### Cognitive function

**Fig. 3:**
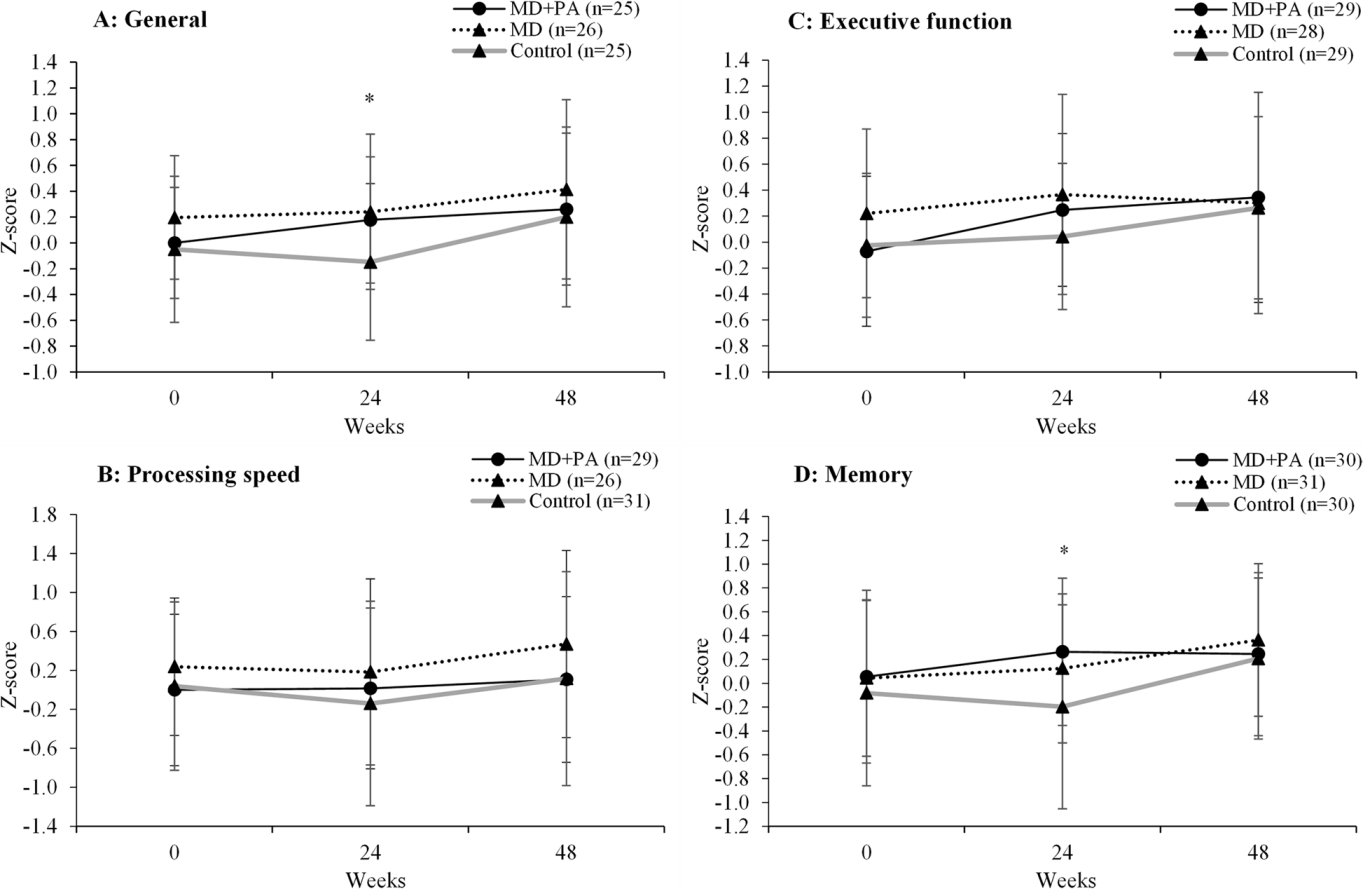
Cognitive summary scores by intervention group at baseline and 24 and 48 weeks. Values represent unadjusted means (SD) for general cognition (**A**), processing speed (**B**), executive function (**C**) and executive function (**D**). * *P*-value < 0.01 for group and contrast 1 (control v. (MD and MD + PA)) at relevant time point compared to baseline, calculated using ANCOVA (adjusted for baseline value, study site baseline age, and years of education). *P*-values for contrast 2 (MD v. MD + PA) were non-significant at all timepoints compared to baseline as were all contrasts comparing values at 48 to 24 weeks. Missing data at 48 weeks were general cognition (MD + PA *n* = 7, MD *n* = 3, control *n* = 8), processing (MD + PA *n* = 5, MD *n* = 5, control *n* = 9), executive function (MD + PA *n* = 6, MD *n* = 10, control *n* = 11) and executive function (MD + PA *n* = 11, MD *n* = 9, control *n* = 8). Individual test scores were converted to *Z* scores standardised on baseline grand mean and standard deviation with response time variables reversed by [*Z* * − 1], so a higher time indicates a better outcome. Individual *Z* scores test scores were mean aggregated into summary scores for the following: Processing speed [Digit symbol substitution (total correct); Trail Making Test (A, seconds)]; Executive Function [Controlled Oral Word Association Test (total); Categorical verbal fluency test (total); Trail Making Test (B-A, seconds); Wechsler Memory Digit Span (backwards, total)] and Memory [Verbal paired immediate (total); Visual paired immediate (total); Verbal paired delayed (total); Visual paired delayed (total); Rey Auditory Verbal Learning Test (immediate); Rey Auditory Verbal Learning Test (recall)]. A general cognition score was calculated using all Processing speed, Executive function and Memory tests. MD, Mediterranean diet; MEDAS, Mediterranean Diet Adherence Screener; PA, physical activity. Full data is presented in Additional file 1: Table S7

After the 24-week intervention, there were improvements in test scores for general cognition (0.22 (95% CI 0.05, 0.35) and memory (0.31 (95% CI 0.10, 0.51) domains in the two MD groups compared with control (Fig. 3 and Additional file 1: Table S7). These changes were determined by improvements observed in the Verbal Paired Associates task, a measure of verbal memory (4.2 (95% CI 0.06, 0.77, Additional file 1: Table S8). There were no significant differences in the test score for the processing speed or executive function domains. Significant differences between the MD + PA and MD groups were not observed for any of the domains, with trends (*P* = 0.06-0.08) for greater performance in the Trail Making, Verbal Paired Associates and Rey Auditory Verbal Learning tests following MD + PA versus MD (Additional file 1: Table S8). Group by sex interactions were not evident for the cognitive summary scores or measures of verbal memory. At 48 weeks, no between-group differences were observed in test scores in any domain.

In the Hayling test of Executive Function, response times reduced for section A (− 5.0 s (95% CI − 7.5, − 2.5)) and section B (− 13.5 s (95% CI − 21.8, − 5.3)) over the 24-week intervention in the two MD groups compared with the control group, with no differences observed in the number of errors made (raw or scaled) (Additional file 1: Table S9). There were no significant differences in response times or number of errors made between groups at 48 weeks (Additional file 1: Table S9). The Hayling overall scaled score improved in the two intervention groups relative to control at 24 weeks (0.7 (95% CI 0.4, 1.0)) but not 48 weeks (Additional file 1: Table S9). Differences between the MD + PA and MD groups were not observed for any of the Hayling outcome measures.

Improvements in test scores in the general cognition (T3–T1 0.29 (95% CI 0.08, 0.50) and memory domains (T3–T1 0.35 (95% CI 0.10, 0.60) over 24 weeks were greater in the participants with the highest change in MEDAS score over the same time (Additional file 1: Table S10). Likewise, there were also improvements in the Hayling test scores with overall scaled scores improved in the participants with the highest change in MEDAS score (T3–T1 1.1 (95% CI 0.3, 1.9)). For physical activity, fewer section B errors were observed in the participants with the greatest increases in moderate activity (T3–T1 − 1.8 (95% CI − 3.3, − 0.2), Additional file 1: Table S11).

### Cardiometabolic outcome

At 24 weeks, there was no significant intervention effect on BMI (Table 2), but at 48 weeks, BMI reduced (− 0.71 kg/m^2^ (95% CI − 1.30, − 0.13)) in the MD + PA and MD groups compared with control. There were no intervention effects on 24-h mean systolic, diastolic or pulse pressure at 24 weeks but a reduction in pulse pressure variability (− 2.9 mm Hg (95% CI − 5.3, − 0.5)) and Ambulatory Arterial Stiffness Index (− 0.07 (95% CI − 0.2, 0.02)) was observed in the MD + PA group in participants with data available. Twenty-four-hour ambulatory blood pressure data were not collected at 48 weeks.

Improvements in pulse pressure variability (T3–T1 − 3.4 (95% CI − 6.1, − 0.7), Additional file 1: Table S10) and Ambulatory Arterial Stiffness Index (T3–T1 − 0.2 (95% CI − 0.3, − 0.1)) over 24 weeks were greater in those participants with the highest change in MEDAS score. No associations were observed between change in physical activity and cardiometabolic outcomes (Additional file 1: Table S11).

### Mechanism of impact measures

At baseline, participants were confident (perceived control) and motivated to change their diet and increase PA. Perceived control and intention reduced over the 24-week intervention period in all groups, with no significant between-group differences observed (Additional file 1: Table S12). Self-reported use of behaviour change techniques taught in the intervention was higher among intervention participants than control participants with goal setting (score 4.5 out of a possible five), and incorporating dietary (score 4.4 out of a possible five) change into daily routines was the most frequently utilised behaviour change technique by intervention participants (with lower levels for PA compared with diet). Conversely, social support (score 3.2 out of a possible five) and self-rewards (score 2.0 out of a possible five) were used least often (Additional file 1: Table S13).

## Discussion

This 24-week multi-domain, theory-informed intervention in older, ‘at risk’ adults living in the UK proved to be feasible and acceptable as judged by our ability to recruit the intended number of completers (with pre-specified characteristics) and the high levels of retention at follow-up. Our ability to deliver the intervention as intended was compromised due to COVID-19 and social distancing restrictions, in particular access to PA opportunities and in-person group sessions. The MedEx-UK intervention was successful in improving eating behaviour (but not PA), with these changes maintained during the 6 months follow-up. A priori, we specified successful eating behaviour change as a 3-point increase on the MEDAS. Participants in the intervention groups achieved a 3.7-point increase in MEDAS at 24 weeks, with a 2.7-point increase maintained at 48-week follow-up. Change in MEDAS scores of this magnitude are likely to be biologically and clinically important with previous studies reporting an approximate 30% reduced risk of major cardiovascular events [44], a 12.6 to 20.7% reduced risk of dementia [11] and up to 5 years of reduced global cognitive ageing [10] with a change in MEDAS score of 2–3 points.

Participants in the current study reported improved adherence to all dietary components (in particular, fish, nuts and olive oil), except for sugar-sweetened beverages which were habitual relatively low at baseline. Conversely, in the PREDIMED study, conducted in a Mediterranean-region, dietary changes were only apparent for foods attributable to the free products provided (olive oil and nuts), legumes and fish [44]. This suggests that more food changes were required by UK participants to align with the MD, but these changes were achievable and maintained for one year.

Between group differences in PA were not observed, with the approximately 70 min per week increase in the MD + PA group at 24 weeks, not reaching significance. This was not entirely unexpected given that the study took place during COVID-19 lockdowns where a wide-range of individual- and group-level activity opportunities, including team sports and indoor facilities, were restricted. It was of interest that activity levels only increased in the MD + PA group and decreased in the other groups which may suggest that the PA component was effective at maintaining activity levels during COVID-19 lockdowns. A recent large US cohort study reported that increasing activity by 10 min per day could reduce preventable deaths by 7% per year [45], and 10-min activity bouts have been linked to improved cognition [46]. Of note, at screening, all participants self-reported < 90-min moderate-intensity PA each week, although at baseline, using directly measured activity, mean moderate activity was 191 min per week in the MD + PA group, with only 34% of the group below the 90-min threshold. This highlights the weaknesses of subjective versus objective PA assessment and suggests refinement of PA methodology at screening is required in future studies, with the use of objective measures of PA wherever possible, to ensure recruitment of intended participants. This may be another factor to explain the moderate changes in PA we observed in the intervention.

Whilst the current study did not observe significant changes in PA, it is notable that the additional behaviour targets in the MD + PA group was not a deterrent to improving eating behaviour and was associated with improvements in select cognitive and cardiometabolic health outcomes. Pulse pressure and ambulatory stiffness index, but not systolic or diastolic blood pressure, measured using 24-h ambulatory blood pressure, were improved only in the MD + PA group at 24 weeks. We also observed a dose effect with greater improvements in cognition and cardiovascular health in participants with the highest levels of behaviour change. Research suggests there are synergistic associations between an individual’s lifestyle risk behaviours and health outcomes which highlights the importance of developing interventions that tackle multiple behavioural risk factors [47].

The intervention was effective at improving general cognition and the composite memory (specifically verbal memory) score over 24 weeks, with the greatest improvements evident in those with the greatest increases in MEDAS score. No intervention effects were observed for processing speed or executive memory. It was unexpected that cognitive improvements were not maintained at 48 weeks which is likely a consequence of the smaller sample size in the maintenance phase (24 to 48 weeks) and may indicate that a longer duration of intervention is required for sustained cognitive benefits. Our findings of improvements in general cognition and memory support those of the FINGER trial with the trend for the same beneficial effects on executive function (with exception of significant effect in the Hayling test), which again may reflect the shorter duration of our intervention [5]. The Hayling processing speed component appeared to be the most sensitive cognitive measure and may be important to explore in future studies. Although the finding should be interpreted with caution due to a small sample size, the significant effect of intervention on vascular stiffness and pulse pressure variability suggest that the cognitive benefits may be in part due to improved cerebrovascular function, with the individual and additive impact of MD bioactives such as wholegrains, dietary fibre, antioxidant vitamins, omega-3 fatty acids and polyphenols on the gut microbiome, neuroinflammation, neurogenesis, glial function, brain hypometabolism, synaptic plasticity and biomarker burden also likely mediating factors [48, 49]. Taking polyphenols and verbal memory as an example, the Supplémentation en Vitamines et Minéraux Antioxydants cohort reported significant associations between total polyphenol intake and language and verbal memory over 13 years [50], with a polyphenol-rich extract from grape and blueberry improving verbal episodic and recognition memory in older adults over 6 months [51]. Preclinical models have identified multiple mechanistic targets which mediate polyphenol-neurophysiological associations including antioxidant, anti-inflammatory and signalling processes and the modulation of synaptic function, cerebral blood flow and gut microbiota speciation and metabolism factors [19, 23, 52].

Engagement with two of the three intervention components designed to support behaviour change was high, specifically the group sessions and uptake of food provision. Conversely, whilst the website was accessible to all participants, use was low and rated poorly. Focus groups highlighted this was mainly due to the poor functionality, which will need to be optimised prior to large-scale evaluation. A previous study in the UK evaluating the feasibility of a peer support intervention to encourage adoption of a MD reported challenges with recruitment and retention of participants [53, 54]. The successful 95% retention in the current study to the primary study endpoint (24 weeks) may be due to the use of individual-level recruitment, rather than the group-based approaches employed in the previous study, which may have ensured the inclusion of more engaged participants. The addition of a food provision component to remove barriers associated with the perceived higher price and inconvenience of healthy foods is also likely to have improved retention. Previous studies have shown that financial support improves adherence to a MD when accompanied by an educational intervention [55]. We provided participants with options to choose MD components that met their personal food preferences, rather than being prespecified by study design, as personalisation has shown to lead to sustained changes in dietary behaviour [56].

Process evaluation was an essential part of this feasibility study and provides us with important insights to inform the development of a larger-scale trial. The intervention recruited a highly motivated sample; participants reported high-levels of perceived control and intention to change their diet and increase physical activity although these reduced over the 24-week period. This may be due to unrealistic optimism at baseline, with participants becoming more realistic over time due to experiences with behaviour change [57]. Alternatively, participants who felt that they already made positive changes to their diet and PA may have been less positive about making further changes, explaining the slightly lower scores at follow-up [58]. In addition, the COVID-19 lockdown and other restrictions may have made behaviour change especially challenging. The behaviour change observed after the intervention was not due to increased perceived control or intention and was more likely due to increasing participants’ use of behaviour change techniques, promoted by the intervention, in their daily lives. The most frequently used techniques (goal setting, building routines) facilitate behaviour maintenance. Participants in the MD + PA reported using these techniques slightly less frequently for PA, than for dietary change, which may have contributed to the differences between observed change in MD and in PA at 24 weeks. In contrast, social support was used least often. Although it was included in the group sessions, the restrictions resulting from the pandemic limited opportunities for social support, especially face-to-face.

Strengths of the current study include (i) the development of intervention components which targeted key influences on behaviour based on the COM-B model and included evidence-based behaviour change techniques, (ii) the robust measurements of feasibility and acceptability, which were informed by Medical Research Council guidance for process evaluation [59], (iii) the use of validated measurement tools for assessing the primary (diet and PA) and secondary (cognition and cardiometabolic) outcomes and (iv) the inclusion of both behavioural and clinical data. The COVID-19 pandemic created unique challenges for the intervention study and restricted our ability to collect the data for our secondary cardiometabolic outcomes. However, our successful experiences around remote delivery of this complex intervention will be invaluable for the design and delivery of future interventions. We defined participants ‘at risk’ of dementia using a scale to monitor risk of cardiovascular disease which does not include assessment of other important risk factors for dementia, including cognitive function and family history. We were not successful in recruiting socio-economically disadvantaged, racially or ethnically diverse participants and the recruitment protocol and study design for a future study will need to be modified to ensure such inclusion. Future trials will need to improve the poor conversion rate from screening to recruitment and to further develop and test intervention tools that are acceptable, feasible and inclusive to our target population, in particular, the online platform. Due to the nature of the trial, including necessarily explaining the potential intervention components for each group to all participants during informed consent, it was not possible to blind either the participants or researchers to group allocation and this may have affected participants behaviour, specifically responses to subjective outcome measures. Other limitations include the lack of an objective PA measurement at screening and the small sample size for our vascular outcomes. In addition, this intervention was not powered to test specific hypotheses, with the findings from our secondary analyses exploratory, and need to be interpreted with caution. Furthermore, our results from the study maintenance phase need to be interpreted with caution as those who consented were those with better dietary behaviours at the end of the main intervention period. This trial did not include a PA only arm, so future studies would be needed to understand the effects of PA alone versus a combined diet and PA intervention.

## Conclusions

The intervention to increase Mediterranean diet adherence and physical activity in older adults at risk of dementia was effective at improving eating behaviour, alone and when increased PA was an additional behavioural target, and was acceptable and feasible. The intervention was also successful in maintaining changes in eating behaviour for up to 12 months, which was likely due to intense early support and investment to achieving long-term change. The changes in eating behaviour were associated with cognitive and cardiovascular benefits especially in the combined Mediterranean-style diet and physical activity intervention group.

## Supplementary Information

Additional file 1: Methods S1-S3. S1 Inclusion and exclusion criteria; S2: LEAP^2^ modifications during the 24–48-week behaviour maintenance phase; S3: Cognitive tests used in the MedEx-UK trial; Figs. S1-S4. S1: Flow chart of participants in the MedEx-UK study; S2: Proportion of participants meeting the criteria for individual Mediterranean Diet Adherence Screener componentsat baseline by intervention group in 86 MedEx-UK participants; S3: Proportion of participants adapting to meet the criteria for individual Mediterranean Diet Adherence Screener componentsat 24 weeks by intervention group in 83 MedEx-UK participants; S4: Participants rating of the overall acceptability of the intervention at 24 weeks by group; Tables S1-S13. S1 Mediterranean Diet Adherence Screenerquestionnaire, criteria for scoring and adaptions made to calculate the score from 24-h recalls; S2: MEDAS score and componentsat baseline and 24 weeks by intervention group in 95 MedEx-UK participants; S3: MEDAS score and componentsat baseline to 24 weeks by intervention group in 83 MedEx-UK participants; S4: MEDAS score and physical activity outcomes at baseline and 24 weeks by intervention group in MedEx-UK participants; analysed using intention to treat analysis; S5: Engagement with MedEx-UK intervention components reported at 24 weeks; S6: Acceptability of the MedEx-UK intervention at 24 weeks; S7: Cognitive summary scores by intervention group at baseline, 24 weeks and 48 weeks by intervention group in 97 MedEx-UK participants; S8: Cognitive test scores by intervention group at baseline, 24 and 48 weeks. at baseline and 24 weeks by intervention group in 97 MedEx-UK participants; S9: Outcomes from the Hayling test at baseline, 24 and 48 weeks by intervention group in 97 MedEx-UK participants; S10: Cognitive and cardiometabolic outcomes at baseline and 24 weeks by tertile of 24-week change in MEDAS score in 93 MedEx-UK participants; S11: Cognitive and cardiometabolic outcomes at baseline and 24 weeks by tertile of 24-week change in moderate activity in 88 MedEx-UK participants; S12: Planned behaviour change at baseline and 24-week by intervention group in 95 MedEx-UK participants; S13: Self-reported use of behaviour change techniques at 24 weeks in 69 MedEx-UK participants.

## Data Availability

The data that support the findings of this study are available from the corresponding author, upon reasonable request.

## Abbreviations

COM-B: Capability, Opportunity, Motivation and Behaviour
QRISK2: Cardiovascular risk score
MEDAS: Mediterranean Diet Adherence Screener
MD: Mediterranean diet
PA: Physical activity
