# Supplementary material for: Effectiveness and feasibility of a theory-informed intervention to improve Mediterranean diet adherence, physical activity and cognition in older adults at risk of dementia: the MedEx-UK randomised controlled trial
Source: BMC Med. 2024 Dec 23;22:600. doi: 10.1186/s12916-024-03815-z (PMC11667912; doi:10.1186/s12916-024-03815-z)
Supplement: Supplementary file 1 — Additional file 1: Methods S1-S3. S1 Inclusion and exclusion criteria; S2: LEAP2 modifications during the 24–48-week behaviour maintenance phase; S3: Cognitive tests used in the MedEx-UK trial; Figs. S1-S4. S1: Flow chart of participants in the MedEx-UK study; S2: Proportion of participants meeting the criteria for individual Mediterranean Diet Adherence Screener componentsat baseline by intervention group in 86 MedEx-UK participants; S3: Proportion of participants adapting to meet the criteria for individual Mediterranean Diet Adherence Screener componentsat 24 weeks by intervention group in 83 MedEx-UK participants; S4: Participants rating of the overall acceptability of the intervention at 24 weeks by group; Tables S1-S13. S1 Mediterranean Diet Adherence Screenerquestionnaire, criteria for scoring and adaptions made to calculate the score from 24-h recalls; S2: MEDAS score and componentsat baseline and 24 weeks by intervention group in 95 MedEx-UK participants; S3: MEDAS score and componentsat baseline to 24 weeks by intervention group in 83 MedEx-UK participants; S4: MEDAS score and physical activity outcomes at baseline and 24 weeks by intervention group in MedEx-UK participants; analysed using intention to treat analysis; S5: Engagement with MedEx-UK intervention components reported at 24 weeks; S6: Acceptability of the MedEx-UK intervention at 24 weeks; S7: Cognitive summary scores by intervention group at baseline, 24 weeks and 48 weeks by intervention group in 97 MedEx-UK participants; S8: Cognitive test scores by intervention group at baseline, 24 and 48 weeks. at baseline and 24 weeks by intervention group in 97 MedEx-UK participants; S9: Outcomes from the Hayling test at baseline, 24 and 48 weeks by intervention group in 97 MedEx-UK participants; S10: Cognitive and cardiometabolic outcomes at baseline and 24 weeks by tertile of 24-week change in MEDAS score in 93 MedEx-UK participants; S11: Cognitive and cardiometabolic outcomes at baseline and 24 weeks [file 12916_2024_3815_MOESM1_ESM.docx]

**Methods S1:** Inclusion and exclusion criteria

**Methods S2:** LEAP^2^ modifications during the 24-48-week behaviour maintenance phase.

**Methods S3:** Cognitive tests used in the MedEx-UK trial

**Figure S1**: Flow chart of participants in the MedEx-UK study

**Figure S2:** Proportion of participants meeting the criteria for individual Mediterranean Diet Adherence Screener components (using questionnaire data) at baseline by intervention group in 86 MedEx-UK participants.

**Figure S3:** Proportion of participants adapting to meet the criteria for individual Mediterranean Diet Adherence Screener components (using 24-hr recall data) at 24-weeks by intervention group in 83 MedEx-UK participants.

**Figure S4:** Participants rating of the overall acceptability of the intervention at 24-weeks by group

**Table S1:** Mediterranean Diet Adherence Screener (MEDAS) questionnaire, criteria for scoring and adaptions made to calculate the score from 24-hr recalls.

**Table S2:** MEDAS score and components (using questionnaire data) at baseline and 24-weeks by intervention group in 95 MedEx-UK participants.

**Table S3:** MEDAS score and components (using 24-hr recall data) at baseline to 24-weeks by intervention group in 83 MedEx-UK participants.

**Table S4:** MEDAS score and physical activity outcomes at baseline and 24-weeks by intervention group in MedEx-UK participants; analysed using intention to treat analysis.

**Table S5:** Engagement with MedEx-UK intervention components reported at 24-weeks

**Table S6:** Acceptability of the MedEx-UK intervention at 24-weeks

**Table S7**: Cognitive summary scores by intervention group at baseline, 24- weeks and 48-weeks by intervention group in 97 MedEx-UK participants.

**Table S8**: Cognitive test scores by intervention group at baseline, 24- and 48- weeks. at baseline and 24-weeks by intervention group in 97 MedEx-UK participants.

**Table S9**: Outcomes from the Hayling test at baseline, 24- and 48-weeks by intervention group in 97 MedEx-UK participants.

**Table S10**: Cognitive and cardiometabolic outcomes at baseline and 24-weeks by tertile of 24-week change in MEDAS score in 93 MedEx-UK participants.

**Table S11**: Cognitive and cardiometabolic outcomes at baseline and 24-weeks by tertile of 24-week change in moderate activity in 88 MedEx-UK participants.

**Table S12:** Planned behaviour change at baseline and 24-week by intervention group in 95 MedEx-UK participants.

**Table S13:** Self-reported use of behaviour change techniques at 24-weeks in 69 MedEx-UK participants.

**Methods S1: Inclusion and exclusion criteria**

Inclusion criteria

- Male and female aged 55 to 74 years.
- QRISK2 score≥10%.
- Stable use of any prescribed medication for at least 4 weeks.
- Understands and is willing and able to comply with all study procedures.
- Has access to, and able to use, the Internet and a computer/tablet.
- Normal, or corrected to normal, vision and hearing.
- Fluent in written and spoken English.

Exclusion criteria

- Diagnosis of Alzheimer’s disease, other forms of dementia, mild cognitive impairment or other significant neurological disorder.
- Cognition not within the normal range, based on a score of less than 23 on the Montreal Cognitive Assessment; or indication of cognitive decline, based on a score of two or more on Ascertain Dementia screening tool.
- Evidence of impairment of Instrumental Activities of Daily Living.
- Moderate-to-severe depression, assessed by the Patient Health Questionnaire.
- Moderate-to-severe anxiety, assessed by the Generalised Anxiety Disorder questionnaire.
- Current psychotic illness (delusional disorder/schizophrenia).
- History of serious mental illness know to affect cognition (schizophrenia, schizoaffective disorder, bipolar disorder).
- Subjects with other clinically diagnosed psychiatric disorders likely to affect the cognitive measures (as judged by a clinical advisor).
- History or previous MRI evidence of brain damage, significant head trauma (including loss of consciousness as a result), brain surgery, stroke or serious neurological disorders.
- Human immunodeficiency virus infection.
- History of alcohol or drug dependency in the last two years.
- Subjects with existing diagnosed gastrointestinal disorders likely to impact study results (as judged by a clinical advisor).
- History of any major cardiovascular event, such as a myocardial infarction, stroke or transient ischaemic attack.
- Severe chronic obstructive pulmonary disease.
- Cancer, or cancer treatment within the last 12 months.
- Diagnosis of Type I or Type II diabetes in the previous three months.
- Clinical diagnosis of liver or kidney disease (level three or above).
- Epilepsy.
- Other medical conditions likely to influence the study measures (as judged by a clinical advisor).
- Body mass index of more than 40 kg/m^2^.
- Habitual Mediterranean Diet Score greater than eight (on the 14-point MEDAS (Mediterranean Diet Adherence Screener) questionnaire).
- Habitual physical activity of more than 90 min moderate activity per week, assessed using the International Physical Activity Questionnaire short form.
- Currently engaged in a weight loss, other dietary or physical activity intervention.
- Currently, a participant or have participated in any other study involving an investigational product in the last four weeks.
- Metal implants, for example, pacemaker that precludes MRI.

**Methods S2: LEAP^2^ modifications during the 24-48-week behaviour maintenance phase**

Participants were given access to a modified version of the LEAP^2^ platform during the 24-48-week behaviour maintenance phase of MedEx-UK, which was designed to help them maintain the healthy lifestyle behaviours established during the initial 0-24-week study intervention period. Modifications included:

**Eating Well module**: Further recipes were added to the Eating Well module to provide additional options to help participants continue adhering to a Mediterranean Diet.

**Moving More module**: Additional activity links were added to the Moving More module to provide continued inspiration for maintaining higher PA levels. This included options for exercising at home (e.g., links to exercise videos), which was deemed particularly important given the ongoing COVID-19 pandemic limited opportunities for exercising outside.

**My Stats module**: An additional LEAP^2^ module was added to help participants prospectively track their Mediterranean Diet adherence and PA levels. This module included graphs which were populated using information provided in the dietary and PA screening questionnaires in the Eating Well and Moving More modules.

**Easier to access content**: Modifications were made to LEAP^2^ to improve navigation and content access, including the addition of drop-down menus for each module.

**Diary feature**: The diary feature of LEAP^2^ was modified to: A) send text or email reminders to participants on the day of their planned meal/activity, B) send follow-up text messages following planned meals/ activities to help encourage participants to identify additional meals/ activities they might enjoy, and C) allow participants to share meals or activities with a friend to increase social support opportunities.

**Methods S3: Cognitive tests used in the MedEx-UK trial**

| **Task** | **Time (minutes)** | **Extended NTB sub-domain** | **Outcome** |
| --- | --- | --- | --- |
| **Part 1** | **45-50** | **-** |  |
| Verbal Paired Associates, immediate | 9-12 | Memory | Total (Sets 1-3) |
| Visual Paired Associates, immediate | 6 | Memory | Total (Sets 1-3) |
| COWAT | 3 | Executive function | Total (Trails FAS) |
| CFT | 1 | Executive function | Total |
| Digit Symbol Substitution | 2 | Processing speed | Total correct |
| TMT A | 2 | Processing speed | Seconds (A) |
| TMT B | 3 | Executive function | Seconds (B-A) |
| Verbal Paired Associates, delayed recall | 5-8 | Memory | Total correct |
| Visual Paired Associates, delayed recall | 6 | Memory | Total correct |
| **BREAK** | **15** | - |  |
| **Part 2** | **45-50** | - |  |
| RAVLT, immediate | 10-15 | Memory | Total (A6) |
| Hayling Sentence Completion | 5-10 | Response inhibition | Time to complete (Section A & B) Errors (Section A & B) |
| Digit Span | 3-5 | Executive function | Total backwards |
| Supermarket Trolley Task | 10 | Spatial navigation |  |
| RAVLT, delayed recall | 3 | Memory | Total (A7) |
| Sea Hero Quest | 10-15 | Spatial navigation |  |

CFT: Category Fluency Test; COWAT: Controlled Oral Word Association Test; RVLT: Rey Auditory Verbal Learning Test; TMT: Trail Making Test.

**Figure S1: Flow chart of participants in the MedEx-UK study**

^1^ Participants may have been excluded for more than one reason but are counted only once.

^2^ Unsuitable = incorrect age, not able to communicate in English, no reliable access to the internet or without normal or corrected vision and/ or hearing. MD = Mediterranean diet, PA = Physical activity

**Completed online screening**

**n= 2776**

**Ineligible**^1^ **(n=2537)**

Unsuitable^2^ n= 234

Health exclusion n= 519

MEDAS score ≥9 points n= 309

Physically active n= 943

Withdrew/ incomplete survey n= 532

**Attended screening**

**n= 239**

**Ineligible**^1^ **(n=135)**

QRISK score <10 n= 91

BMI >40 kg/m^2^ n= 2

Mild cognitive impairment n= 9

Depression n= 3

Anxiety n= 3

Without independent living skills n= 3

Withdrew/ incomplete survey n= 24

**Randomly assigned & completed baseline assessment**

**n= 104**

MD+PA group = 35

MD group = 35

Control group = 34

**Completed 24-week follow up assessment**

**n= 99**

MD+PA group = 33

MD group = 34

Control group = 32

**Completed 24 to 48-week behaviour maintenance phase assessment add-on**

**n= 69**

MD+PA group = 24

MD group = 23

Control group = 22

**Figure S2: Proportion of participants meeting the criteria for individual Mediterranean Diet Adherence Screener components (using questionnaire data) at baseline by intervention group in 86 MedEx-UK participants.**

Bars represent the percentage of participants who met the criteria at baseline according to the questionnaire data. Only participants with complete data for all components were included (n=86).

**Figure S3: Proportion of participants adapting to meet the criteria for individual Mediterranean Diet Adherence Screener components (using 24-hr recall data) at 24-weeks by intervention group in 83 MedEx-UK participants.**

Bars represent the percentage of participants who met the criteria at 24-weeks but not at baseline according to the 24-hr recall data. Only participants with complete data for all components were included (n=83). Missing bars indicate the percentage of participants was zero.

**Figure S4:** **Participants rating of the overall acceptability of the intervention at 24-weeks by group**

Bars represent the percentage of participants who reported each response at 24-weeks (n=64). Data on individual constructs are presented in Table S6. Missing bars indicate the percentage of participants was zero.

**Table S1: Mediterranean Diet Adherence Screener (MEDAS) questionnaire, criteria for scoring and adaptions made to calculate the score from 24-hr recalls.**

| **Question** | **Criteria for 1 point (g)** | **Criteria for 1 point (portions)** | **Adaptations for calculation from 24-hr recalls** |
| --- | --- | --- | --- |
| 1. Do you use olive oil as the principal source of fat for cooking? | Yes | Yes | Data used from the MEDAS questionnaire |
| 2. How much olive oil do you consume per day (including that used in frying, salads, meals eaten away from home, etc.)? | ≥ 52/ day | ≥ 4 tablespoons/ day | If olive oil was reported as the main source of fat, all liquid fat was assumed to be olive oil. |
| 3. How many servings of vegetables do you consume per day? | ≥ 400/ day | ≥ 2/ day |  |
| 4. How many portions of fruit do you consume per day? (1 serving is 80 g). | ≥ 240/ day | ≥ 3/ day |  |
| 5. How many servings of red meat, hamburgers, or sausages do you consume per day? (1 serving is 100–150 g). | ≤ 125/ day | ≤ 1/ day |  |
| 6. How many servings of butter, margarine, or cream do you consume per day? (1 serving is 12 g) | ≤ 12/ day | ≤ 1/ day |  |
| 7. How many carbonated and/or sugar-sweetened beverages do you consume per day, excluding ‘diet’ drinks? (1 serving is 100 ml) | ≤ 100/ day | ≤ 1/ day |  |
| 8. Do you drink wine? How much do you consume per week? (1 serving is 125 ml). | ≥ 125 /day | ≥ 7/ week |  |
| 9. How many servings of pulses do you consume per week? (1 serving is 150g) | ≥ 64/ day | ≥ 3/ week |  |
| 10. How many servings of fish/seafood do you consume per week? (1 serving is 100–150 g of fish or 200 g of seafood) | ≥ 54/ day | ≥ 3/ week |  |
| 11. How many times do you consume commercial (not homemade) sweets or pastries or ice cream per week? | < 0.3/ day | < 2/ week | It was not possible to determine if products were commercial or homemade, so all were considered. Portion size was not accounted for; any occurrence counted towards the score. |
| 12. How many times do you consume nuts per week? (1 serving = 30 g) | ≥ 13/ day | ≥ 3/ week |  |
| 13. Do you prefer to eat poultry more than red or processed meat? | Yes | Yes |  |
| 14. How many times per week do you consume a sauce of tomato, garlic, onion, or leeks sautéed in olive oil? | ≥ 0.3/ day | ≥ 2/ week | Counted occurrences of mixed dishes likely to contain sofrito, such bolognaise sauce etc, and where tomatoes were listed with onion/ garlic or leek in the same meal. Occurrences only counted if participants reported olive oil as their main source of fat. |

**Table S2: MEDAS score and components (using questionnaire data) at baseline and 24-weeks by intervention group in 95 MedEx-UK participants.**

|  | **MD+PA (n=30)** | | **MD (n=34)** | | **Control (n=31)** | | **P1** | **P2** | **P3** |
| --- | --- | --- | --- | --- | --- | --- | --- | --- | --- |
|  | **Baseline** | **24-weeks** | **Baseline** | **24-weeks** | **Baseline** | **24-weeks** |  |  |  |
| MEDAS total score (maximum score 14) | 6.7 (2.2) | 11.4 (1.8) | 5.9 (2.0) | 10.0 (1.9) | 6.8 (2.1) | 7.2 (2.1) | <0.01 | <0.01 | 0.06 |
| Olive oil (main source fat, y/n^1^) | 0.6 (0.5) | 1.0 (0.0) | 0.5 (0.5) | 1.0 (0.0) | 0.6 (0.5) | 0.8 (0.4) | <0.01 | <0.01 | 0.77 |
| Olive oil (tbsp/ d) | 0.9 (1.0) | 4.0 (2.0) | 1.0 (1.1) | 3.6 (1.8) | 1.0 (1.3) | 2.0 (2.0) | <0.01 | <0.01 | 0.76 |
| Vegetables (200 g servings/d) | 1.9 (0.8) | 3.1 (0.9) | 2.2 (1.0) | 2.6 (1.4) | 2.1 (1.2) | 2.5 (1.1) | 0.02 | 0.14 | 0.01 |
| Fruit (80 g servings/d) | 2.2 (1.2) | 2.9 (1.0) | 1.8 (1.0) | 2.6 (0.8) | 2.3 (1.3) | 2.2 (1.5) | <0.01 | <0.01 | 0.50 |
| Red meat (125 g servings/week) | 3.0 (2.6) | 1.2 (1.1) | 3.6 (3.7) | 1.8 (1.7) | 3.6 (3.8) | 2.6 (1.9) | <0.01 | <0.01 | 0.31 |
| Butter, cream (12 g servings/week) | 5.9 (4.0) | 3.2 (3.6) | 7.2 (6.0) | 3.2 (3.0) | 6.6 (4.9) | 6.6 (5.3) | <0.01 | <0.01 | 0.71 |
| Sugar-sweetened drinks (100 ml servings/week) | 0.9 (2.7) | 1.1 (2.7) | 2.4 (8.0) | 0.2 (0.7) | 0.8 (1.8) | 0.7 (1.6) | 0.34 | 0.73 | <0.01 |
| Wine (125 ml servings/week) | 4.5 (7.0) | 5.2 (3.3) | 3.5 (4.9) | 4.1 (3.5) | 3.1 (5.4) | 2.5 (3.6) | <0.01 | <0.01 | 0.61 |
| Pulses (150 g servings/week) | 2.6 (1.1) | 3.5 (1.3) | 2.2 (1.4) | 3.2 (2.5) | 3.1 (1.4) | 2.4 (1.6) | 0.01 | <0.01 | 0.85 |
| Fish (125 g servings/week) | 2.0 (1.6) | 3.5 (1.6) | 1.9 (1.1) | 3.3 (1.5) | 1.8 (1.2) | 1.9 (1.5) | <0.01 | <0.01 | 0.81 |
| Sweets and pastries (servings/week) | 5.0 (4.4) | 2.0 (2.1) | 5.1 (4.4) | 2.2 (2.6) | 4.9 (5.5) | 3.8 (3.0) | <0.01 | <0.01 | 0.87 |
| Nuts (30 g servings/week) | 2.3 (2.4) | 5.1 (2.6) | 1.5 (2.3) | 3.4 (2.1) | 1.8 (2.0) | 2.3 (2.0) | <0.01 | <0.01 | 0.01 |
| Ratio white/ red meat (y/n^1^) | 0.7 (0.4) | 0.9 (0.3) | 0.7 (0.5) | 1.0 (0.3) | 0.7 (0.5) | 0.7 (0.5) | <0.01 | <0.01 | 0.55 |
| Sofrito (servings/week) | 1.7 (1.2) | 3.5 (1.1) | 1.6 (1.4) | 2.5 (1.2) | 1.5 (1.6) | 2.0 (1.9) | <0.01 | <0.01 | <0.01 |

Values are unadjusted means (SD). P1 = p-value for group using ANCOVA (adjusted for baseline value, study site and baseline BMI), P2 = p-value for contrast 1: Control v. (MD + MDPA), P3 = p-value for contrast 2: MD v. MDPA.

^1^ Data was coded as No=0 and yes=1

Missing data: Olive oil (tbsp/ d) n=3 (n=2 MD, n=1 control); red meat n=2 (n=1 MD+PA, n=1 MD); butter, cream n=1 (MD+PA); sugar-sweetened drinks n=2 (n=1 MD, n=1 control); wine n=1 (MD); pulses n=1 (MD); nuts n=2 (n=1 MD, n=1 control); sofrito n=1 (MD). MEDAS total score uses an adjusted score which accounts for missing data as (total score/ number of components with complete data) *14. MD = Mediterranean diet, MEDAS = Mediterranean Diet Adherence Screener, PA = Physical activity.

**Table S3: MEDAS score and components (using 24-hr recall data) at baseline to 24-weeks by intervention group in 83 MedEx-UK participants.**

|  | **MD+PA (n=30)** | | **MD (n=25)** | | **Control (n=28)** | | **P1** | **P2** | **P3** |
| --- | --- | --- | --- | --- | --- | --- | --- | --- | --- |
|  | **Baseline** | **24-weeks** | **Baseline** | **24-weeks** | **Baseline** | **24-weeks** |  |  |  |
| MEDAS total score (maximum score 14) | 4.7 (1.7) | 8.1 (2.6) | 4.8 (1.3) | 8.3 (2.1) | 4.9 (1.8) | 4.8 (1.8) | <0.01 | <0.01 | 0.68 |
| Vegetables (200 g servings/d) | 1.0 (0.7) | 2.1 (1.1) | 1.2 (0.8) | 2.2 (1.3) | 1.1 (0.7) | 1.4 (1.6) | 0.01 | 0.02 | 0.46 |
| Fruit (80 g servings/d) | 1.9 (1.0) | 3.4 (2.0) | 1.5 (1.2) | 3.1 (1.5) | 2.2 (2.0) | 2.3 (1.7) | <0.01 | <0.01 | 0.80 |
| Red meat (125 g servings/week) | 2.2 (2.0) | 1.1 (1.5) | 2.9 (2.1) | 1.3 (1.4) | 2.3 (2.4) | 2.4 (2.2) | <0.01 | <0.01 | 0.89 |
| Butter, cream (12 g servings/week) | 2.8 (6.5) | 3.9 (8.7) | 3.1 (8.4) | 1.5 (3.6) | 4.1 (13.8) | 3.5 (7.9) | 0.68 | 0.77 | 0.21 |
| Sugar-sweetened drinks (100 ml servings/week) | 4.5 (10.3) | 4.7 (9.4) | 4.1 (6.4) | 2.1 (5.7) | 3.7 (7.0) | 4.2 (7.2) | 0.88 | 0.55 | 0.20 |
| Wine (125 ml servings/week) | 4.0 (6.5) | 6.6 (6.6) | 4.2 (6.1) | 5.9 (5.7) | 3.9 (8.4) | 2.8 (4.2) | 0.02 | <0.01 | 0.93 |
| Red wine (125 ml servings/week) | 1.4 (2.8) | 4.0 (4.9) | 1.7 (3.3) | 4.3 (5.7) | 1.9 (4.7) | 1.8 (3.5) | 0.07 | 0.02 | 0.66 |
| Pulses (150 g servings/week) | 1.0 (1.4) | 1.6 (1.6) | 0.7 (0.9) | 1.9 (2.0) | 0.9 (1.0) | 0.7 (0.9) | 0.04 | <0.01 | 0.26 |
| Fish (125 g servings/week) | 1.9 (1.8) | 3.7 (2.6) | 1.5 (1.5) | 2.9 (1.8) | 1.6 (1.5) | 1.2 (1.0) | <0.01 | <0.01 | 0.27 |
| Sweets and pastries (g/d) | 59.8 (45.4) | 28.1 (34.1) | 59.7 (51.7) | 33.5 (47.5) | 83.2 (66.2) | 76.7 (59.8) | <0.01 | <0.01 | 0.57 |
| Nuts (30 g servings/week) | 2.7 (4.4) | 5.5 (6.2) | 1.9 (2.8) | 5.8 (8.2) | 1.5 (2.1) | 0.9 (1.2) | 0.01 | <0.01 | 0.49 |
| Ratio white/ red meat (y/n^1^) | 0.3 (0.5) | 0.7 (0.4) | 0.4 (0.5) | 0.6 (0.5) | 0.4 (0.5) | 0.4 (0.5) | 0.01 | 0.02 | 0.37 |
| Sofrito (meals/week) | 0.9 (1.4) | 2.7 (1.6) | 0.9 (1.4) | 2.4 (1.8) | 0.8 (1.3) | 1.2 (1.5) | <0.01 | <0.01 | 0.61 |

Values are unadjusted means (SD). P1 = p-value for group using ANCOVA (adjusted for baseline value, study site and baseline BMI), P2 = p-value for contrast 1: Control v. (MD + MDPA), P3 = p-value for contrast 2: MD v. MDPA.

MD = Mediterranean diet, MEDAS = Mediterranean Diet Adherence Screener, PA = Physical activity.

**Table S4: MEDAS score and physical activity outcomes at baseline and 24-weeks by intervention group in MedEx-UK participants; analysed using intention to treat analysis.**

|  | **MD+PA** | | | **MD** | | | **Control** | | | **P1** | **P2** | **P3** |
| --- | --- | --- | --- | --- | --- | --- | --- | --- | --- | --- | --- | --- |
|  | **n=** | **Baseline** | **24-weeks** | **n=** | **Baseline** | **24-weeks** | **n=** | **Baseline** | **24-weeks** |  |  |  |
| MEDAS total score (questionnaire data) | 35 | 6.8 (2.2) | 10.7 (2.3) | 35 | 5.9 (2.0) | 9.9 (2.1) | 34 | 6.8 (2.1) | 7.2 (2.1) | <0.01 | <0.01 | 0.39 |
| MEDAS total score (recall data) | 31 | 4.7 (1.6) | 7.9 (2.7) | 27 | 4.6 (1.4) | 7.9 (2.5) | 30 | 5.0 (1.8) | 4.9 (1.7) | <0.01 | <0.01 | 0.97 |
| Total Steps, d | 34 | 5689 (2091) | 6187 (3165) | 35 | 6216 (2335) | 5882 (2473) | 32 | 6305 (2524) | 5948 (2643) | 0.15 | 0.27 | 0.32 |
| Energy expenditure, kcal/week | 34 | 296 (208) | 334 (306) | 35 | 320 (180) | 280 (147) | 32 | 301 (185) | 240 (128) | 0.04 | 0.10 | 0.20 |
| Moderate activity, min/week | 34 | 181 (154) | 240 (177) | 35 | 230 (205) | 226 (188) | 32 | 261 (308) | 220 (210) | 0.22 | 0.25 | 0.64 |

Values are unadjusted means (SD). P1 = p-value for group using ANCOVA (adjusted for baseline value, study site and baseline BMI), P2 = p-value for contrast 1: Control v. (MD + MDPA), P3 = p-value for contrast 2: MD v. MDPA. Data analysed using intention to treat analysis with baseline values carried forward (n=104; MD+PA n=35; MD n=35; Control n=34).

MD = Mediterranean diet, MEDAS = Mediterranean Diet Adherence Screener, PA = Physical activity.

**Table S5: Engagement with MedEx-UK intervention components reported at 24-weeks**

| **Category** | **All**  **(n=70)** | **MD-PA**  **(n=35)** | **MD**  **(n=35)** | **P-value** |
| --- | --- | --- | --- | --- |
| Self-reported average use of the online platform during 24-week intervention^1^ |  |  |  |  |
| Daily, n (%) | 0 (0 %) | 0 (0 %) | 0 (0 %) | - |
| 2-3 times per week, n (%) | 1 (1.7 %) | 1 (3.3 %) | 0 (0 %) | - |
| Once per week, n (%) | 0 (0 %) | 0 (0 %) | 0 (0 %) | - |
| Once per fortnight, n (%) | 8 (13.9 %) | 3 (10.0 %) | 5 (17.9 %) | - |
| Once per month, n (%) | 26 (44.9 %) | 13 (43.3 %) | 13 (46.4 %) | - |
| Less than once per month, n (%) | 23 (39.5 %) | 13 (43.3 %) | 10 (35.7 %) | 0.61 |
| Minutes per session^2^, mean (SD) | 23.0 (14.4) | 26.3 (15.3) | 19.4 (12.8) | 0.07 |
|  |  |  |  |  |
| Facilitator reported group session attendance |  |  |  |  |
| Zero sessions, n (%) | 1 (1 %) | 1 (3 %) | 0 (0 %) | - |
| One session, n (%) | 2 (3 %) | 0 (0 %) | 2 (6 %) | - |
| Two sessions, n (%) | 7 (10 %) | 4 (11 %) | 3 (9 %) | - |
| Three sessions, n (%) | 10 (14 %) | 6 (17 %) | 4 (11 %) | - |
| Four sessions, n (%) | 50 (71 %) | 24 (69 %) | 26 (74 %) | - |
| Number attended, mean (SD) | 3.5 (0.9) | 3.5 (0.9) | 3.5 (0.9) | 0.79 |

Values are n (%) or mean (SD) where indicated. P-value for difference between groups using independent-sample t-test or χ² test for categorial data.^1^MD+PA missing data n=5, MD missing data for n=7. ^2^Participants were asked to choose the category that indicated how long, on average, they spent on the online platform per session (0 seconds to 5 minutes; 5 to 15 minutes; 15 to 30 minutes; 30 minutes to 1 hour; more than 1 hour) the midpoint of the category was taken as the average time in minutes.

**Table S6: Acceptability of the MedEx-UK intervention at 24-weeks**

|  | **All**  **(n=70)** | **MD-PA**  **(n=35)** | **MD**  **(n=35)** | **P-value** |
| --- | --- | --- | --- | --- |
| How acceptable was the intervention? | 4.4 (0.9) | 4.3 (1.0) | 4.4 (0.8) | 0.49 |
| Did you like or dislike the intervention? | 4.3 (0.8) | 4.4 (0.7) | 4.2 (0.8) | 0.25 |
| How much effort did it take to engage with the LEAP 2 website? | 3.2 (1.1) | 3.1 (1.0) | 3.2 (1.1) | 0.81 |
| How much effort did it take to engage with the group sessions? | 4.0 (1.1) | 3.8 (1.2) | 4.1 (1.0) | 0.27 |
| How much effort did it take to engage with the food ordering system? | 3.8 (0.9) | 3.7 (1.0) | 3.8 (0.8) | 0.69 |
| The intervention fits with my beliefs about healthy eating (and physical activity)? | 4.3 (0.7) | 4.6 (0.6) | 4.1 (0.7) | <0.01 |
| The intervention is likely to change my diet? | 4.2 (0.7) | 4.2 (0.9) | 4.3 (0.6) | 0.64 |
| It is clear to me how the intervention would change my diet (and increase my physical activity levels)? | 4.3 (0.6) | 4.4 (0.7) | 4.2 (0.6) | 0.17 |
| How confident do you feel about eating a Mediterranean diet (and becoming more physically active)? | 4.1 (0.8) | 4.2 (0.8) | 4.1 (0.9) | 0.46 |
| Taking part in the intervention interfered with my other priorities? | 3.7 (1.1) | 4.0 (1.0) | 3.5 (1.1) | 0.04 |

Values are mean (SD) responses on a Likert scale from one to five, where higher numbers indicate a more positive response. P-value for group using ANCOVA (adjusted for study site). Missing data; ^1^ n=1. MD = Mediterranean diet, PA = Physical activity.

**Table S7: Cognitive summary scores by intervention group at baseline, 24- weeks and 48-weeks by intervention group in 97 MedEx-UK participants.**

|  | **MD+PA** | | | **MD** | | | **Control** | | | **P1** | **P2** | **P3** |
| --- | --- | --- | --- | --- | --- | --- | --- | --- | --- | --- | --- | --- |
|  | **n=** | **Baseline** | **24-weeks** | **n=** | **Baseline** | **24-weeks** | **n=** | **Baseline** | **24-weeks** |  |  |  |
| General cognition | 25 | 0.00 (0.4) | 0.18 (0.5) | 21 | 0.20 (0.5) | 0.24 (0.6) | 26 | -0.05 (0.6) | -0.15 (0.6) | <0.01 | 0.01 | 0.21 |
| Processing speed | 29 | 0.00 (0.8) | 0.02 (0.8) | 26 | 0.24 (0.7) | 0.18 (1.0) | 31 | 0.04 (0.9) | -0.14 (1.0) | 0.21 | 0.23 | 0.72 |
| Executive function | 29 | -0.07 (0.6) | 0.25 (0.6) | 28 | 0.22 (0.6) | 0.37 (0.8) | 29 | -0.03 (0.6) | 0.04 (0.6) | 0.05 | 0.08 | 0.45 |
| Memory | 30 | 0.06 (0.7) | 0.26 (0.6) | 31 | 0.04 (0.7) | 0.13 (0.6) | 30 | -0.08 (0.8) | -0.20 (0.9) | <0.01 | 0.00 | 0.24 |
|  |  |  | **48-weeks** |  |  | **48-weeks** |  |  | **48-weeks** |  |  |  |
| General cognition | 18 | -0.20 (0.5) | 0.26 (0.6) | 18 | 0.08 (0.7) | 0.41 (0.7) | 18 | 0.04 (0.7) | 0.20 (0.7) | 0.07 | 0.08 | 0.55 |
| Processing speed | 24 | -0.19 (0.7) | 0.11 (0.9) | 21 | -0.04 (1.0) | 0.47 (1.0) | 22 | 0.09 (0.9) | 0.12 (1.1) | 0.39 | 0.13 | 0.40 |
| Executive function | 23 | -0.16 (0.6) | 0.34 (0.8) | 18 | 0.09 (0.8) | 0.30 (0.9) | 18 | 0.06 (0.7) | 0.26 (0.7) | 0.20 | 0.44 | 0.27 |
| Memory | 19 | -0.07 (0.6) | 0.25 (0.7) | 22 | 0.00 (0.7) | 0.36 (0.6) | 22 | -0.04 (0.8) | 0.21 (0.7) | 0.50 | 0.34 | 0.73 |

Values are unadjusted means (SD). P1 = p-value for group using ANCOVA (adjusted for baseline value, study site baseline age, and years of education), P2 = p-value for contrast 1: Control v. (MD & MD+PA), P3 = p-value for contrast 2: MD v. MD+PA. Individual test scores were converted to Z scores standardised on Baseline grand mean and standard deviation with response time variables reversed by [Z * -1], so a higher time indicates a better outcome. Individual Z scores test scores were mean aggregated into Summary Scores for: Processing speed [Digit symbol substitution (total correct); Trail Making Test (A, seconds)]; Executive Function [Controlled Oral Word Association Test (total); Categorical verbal fluency test (total); Trail Making Test (B-A, seconds); Wechsler Memory Digit Span (backwards, total)] and Memory [Verbal paired immediate (total); Visual paired immediate (total); Verbal paired delayed (total); Visual paired delayed (total); Rey Auditory Verbal Learning Test (immediate); Rey Auditory Verbal Learning Test (recall)]. A general cognition score was calculated using all Processing speed, Executive function and Memory tests.

MD = Mediterranean diet, PA = Physical activity.

**Table S8: Cognitive test scores by intervention group at baseline, 24- and 48- weeks. at baseline and 24-weeks by intervention group in 97 MedEx-UK participants.**

|  | **MD+PA** | | | **MD** | | | **Control** | | | **P1** | **P2** | **P3** |
| --- | --- | --- | --- | --- | --- | --- | --- | --- | --- | --- | --- | --- |
|  | **n=** | **Baseline** | **24-weeks** | **n=** | **Baseline** | **24-weeks** | **n=** | **Baseline** | **24-weeks** |  |  |  |
| COWAT (total) | 32 | -0.01 (0.9) | 0.12 (1.0) | 33 | 0.17 (0.9) | 0.46 (0.9) | 32 | -0.02 (1.1) | -0.06 (0.8) | 0.23 | 0.03 | 0.19 |
| CFT (total) | 32 | -0.13 (0.9) | 0.25 (1.0) | 33 | 0.07 (1.2) | 0.32 (1.1) | 32 | 0.15 (1.0) | 0.10 (1.0) | 0.09 | 0.07 | 0.76 |
| TMT (B-A, seconds) | 30 | -0.24 (1.1) | 0.31 (0.6) | 28 | 0.38 (0.9) | 0.12 (1.5) | 29 | 0.04 (0.9) | 0.08 (0.8) | 0.17 | 0.56 | 0.08 |
| WMDS (backwards, total) | 31 | 0.05 (0.8) | 0.15 (1.0) | 33 | 0.17 (1.2) | 0.37 (0.9) | 32 | -0.08 (1.1) | 0.22 (1.1) | 0.70 | 0.90 | 0.57 |
| DSS (total correct) | 29 | 0.02 (0.9) | 0.02 (0.9) | 27 | 0.20 (1.0) | 0.06 (1.3) | 31 | -0.08 (1.1) | -0.08 (1.1) | 0.94 | 0.83 | 0.60 |
| TMT (A, seconds) | 32 | -0.09 (1.1) | 0.01 (1.1) | 32 | 0.12 (0.9) | 0.28 (0.9) | 32 | 0.13 (0.9) | -0.18 (1.2) | 0.16 | 0.05 | 0.54 |
| VPA immediate (total) | 32 | 0.08 (1.0) | 0.69 (0.7) | 33 | 0.10 (0.9) | 0.32 (0.9) | 32 | -0.20 (1.1) | -0.01 (1.0) | <0.01 | 0.02 | 0.07 |
| VPC immediate (total) | 32 | 0.01 (0.8) | 0.43 (1.2) | 32 | -0.09 (1.2) | 0.53 (0.8) | 31 | 0.11 (1.0) | 0.08 (1.3) | 0.12 | 0.04 | 0.59 |
| VPA delayed (total) | 32 | -0.15 (1.1) | 0.09 (0.8) | 33 | 0.16 (0.8) | 0.36 (0.8) | 32 | -0.07 (1.1) | -0.20 (0.9) | 0.07 | 0.01 | 0.38 |
| VPC delayed (total) | 31 | -0.04 (1.1) | 0.10 (0.8) | 32 | 0.01 (0.9) | 0.33 (0.3) | 31 | -0.04 (1.2) | 0.03 (1.1) | 0.55 | 0.21 | 0.32 |
| RVLT (immediate) | 31 | 0.17 (1.0) | -0.05 (1.2) | 32 | 0.09 (1.0) | -0.45 (1.3) | 31 | -0.23 (1.0) | -0.50 (1.2) | 0.49 | 0.98 | 0.15 |
| RVLT (recall) | 32 | 0.10 (1.0) | 0.09 (1.1) | 33 | 0.02 (0.9) | -0.33 (1.1) | 31 | -0.12 (1.0) | -0.46 (1.1) | 0.05 | 0.22 | 0.06 |
|  |  |  | **48-weeks** |  |  | **48-weeks** |  |  | **48-weeks** |  |  |  |
| COWAT (total) | 23 | -0.13 (1.0) | 0.33 (1.0) | 22 | 0.11 (0.8) | 0.56 (0.9) | 22 | -0.04 (1.2) | 0.04 (1.2) | 0.11 | 0.06 | 0.92 |
| CFT (total) | 24 | -0.10 (0.9) | 0.26 (1.2) | 22 | -0.09 (1.0) | 0.16 (0.9) | 22 | 0.14 (1.0) | 0.53 (0.9) | 0.67 | 0.44 | 0.64 |
| TMT (B-A, seconds) | 23 | -0.28 (1.2) | 0.29 (0.7) | 18 | 0.15 (1.2) | 0.02 (1.2) | 18 | 0.09 (1.0) | 0.00 (0.9) | 0.11 | 0.30 | 0.20 |
| WMDS (backwards, total) | 24 | -0.11 (0.7) | 0.44 (1.1) | 22 | -0.07 (1.2) | 0.34 (1.3) | 22 | -0.09 (1.0) | 0.05 (1.2) | 0.19 | 0.24 | 0.60 |
| DSS (total correct) | 24 | -0.16 (0.8) | 0.12 (1.0) | 22 | 0.10 (1.1) | 0.75 (1.2) | 22 | 0.02 (1.0) | 0.11 (1.4) | 0.55 | 0.16 | 0.23 |
| TMT (A, seconds) | 24 | -0.23 (1.1) | 0.09 (1.0) | 21 | -0.14 (1.1) | 0.21 (0.9) | 22 | 0.15 (0.9) | 0.12 (1.0) | 0.60 | 0.51 | 0.93 |
| VPA immediate (total) | 24 | 0.08 (1.0) | 0.85 (0.9) | 22 | 0.07 (1.0) | 1.02 (0.8) | 22 | -0.10 (1.0) | 0.63 (0.6) | 0.38 | 0.18 | 0.59 |
| VPC immediate (total) | 24 | -0.07 (0.8) | 0.79 (0.7) | 22 | -0.28 (1.2) | 0.92 (0.6) | 22 | 0.16 (1.1) | 0.55 (1.1) | 0.05 | 0.00 | 0.28 |
| VPA delayed (total) | 24 | -0.20 (1.1) | 0.15 (0.9) | 22 | 0.12 (0.9) | 0.46 (0.6) | 22 | 0.01 (1.1) | 0.50 (0.7) | 0.17 | 0.37 | 0.26 |
| VPC delayed (total) | 24 | 0.07 (0.9) | 0.31 (0.3) | 22 | -0.07 (0.9) | 0.14 (0.6) | 22 | -0.22 (1.4) | -0.07 (1.0) | 0.08 | 0.08 | 0.72 |
| RVLT (immediate) | 19 | 0.09 (1.0) | -0.10 (1.2) | 22 | 0.12 (1.1) | -0.22 (1.1) | 22 | -0.07 (1.0) | -0.18 (0.9) | 1.00 | 0.81 | 0.66 |
| RVLT (recall) | 19 | -0.12 (1.0) | -0.31 (1.2) | 22 | 0.06 (1.0) | -0.15 (1.1) | 22 | -0.04 (1.0) | -0.18 (1.0) | 0.98 | 0.93 | 0.83 |

Values are unadjusted means (SD). P1 = p-value for group using ANCOVA (adjusted for baseline value, study site baseline age, and years of education), P2 = p-value for contrast 1: Control v. (MD & MD+PA), P3 = p-value for contrast 2: MD v. MD+PA. Individual test scores were converted to Z scores standardised on Baseline grand mean and standard deviation with response time variables reversed by [Z * -1], so a higher time indicates a better outcome. CFT= Category Fluency Test; COWAT= Controlled Oral Word Association Test; DSS = Digit Symbol Substitution; MD = Mediterranean diet, PA = Physical activity; RVLT= Rey Auditory Verbal Learning Test; TMT= Trail Making Test; VPA= Verbal Paired Associates; VPC= Visual Paired Associate; WMDS = Wechsler Memory Digit Span.

**Table S9: Outcomes from the Hayling test at baseline, 24- and 48-weeks by intervention group in 97 MedEx-UK participants.**

|  | **MD+PA (n=32)** | | **MD (n=33)** | | **Control (n=32)** | | **P1** | **P2** | **P3** |
| --- | --- | --- | --- | --- | --- | --- | --- | --- | --- |
|  | **Baseline** | **24-weeks** | **Baseline** | **24-weeks** | **Baseline** | **24-weeks** |  |  |  |
| Section A, time, seconds | 6.8 (7.5) | 9.5 (6.6) | 4.5 (3.7) | 9.8 (6.5) | 5.4 (4.7) | 14.3 (7.9) | <0.01 | <0.01 | 0.18 |
| Section A, time, scaled | 5.8 (0.9) | 5.5 (0.8) | 5.9 (0.5) | 5.5 (0.8) | 5.8 (0.5) | 4.9 (1.0) | <0.01 | <0.01 | 0.49 |
| Section B, time, seconds | 32.7 (25.6) | 30.5 (19.9) | 43.5 (23.4) | 31.5 (18.7) | 27.0 (20.6) | 38.3 (28.2) | 0.03 | <0.01 | 0.30 |
| Section B, time, scaled | 5.6 (0.9) | 5.8 (0.7) | 5.4 (1.0) | 5.8 (0.4) | 5.8 (0.7) | 5.5 (1.1) | 0.07 | <0.01 | 0.28 |
| Category A errors, total | 0.6 (0.8) | 0.3 (0.5) | 0.7 (1.2) | 0.4 (0.6) | 0.7 (0.9) | 0.6 (0.8) | 0.15 | 0.15 | 0.69 |
| Category A errors, scaled | 1.9 (2.7) | 1.0 (1.6) | 2.3 (4.6) | 1.3 (1.8) | 2.1 (2.8) | 1.8 (2.3) | 0.14 | 0.14 | 0.72 |
| Category B errors, total | 1.8 (1.6) | 1.4 (1.5) | 2.2 (1.7) | 1.2 (1.3) | 1.5 (2.2) | 1.8 (2.1) | 0.16 | 0.05 | 0.54 |
| Category B errors, scaled | 2.3 (3.3) | 1.8 (2.8) | 2.8 (3.4) | 1.2 (1.3) | 2.8 (6.0) | 2.9 (4.8) | 0.15 | 0.04 | 0.49 |
| Total errors, scaled | 6.4 (1.2) | 6.8 (1.1) | 6.3 (1.3) | 6.9 (0.8) | 6.4 (1.9) | 6.3 (1.6) | 0.09 | 0.03 | 0.74 |
| Total scaled score | 17.8 (1.9) | 18.0 (1.8) | 17.7 (1.9) | 18.2 (1.5) | 18.1 (2.2) | 16.7 (2.3) | <0.01 | <0.01 | 0.62 |
| Overall scaled score | 5.9 (0.9) | 5.9 (0.8) | 5.8 (0.9) | 6.1 (0.7) | 6.0 (1.1) | 5.4 (0.9) | <0.01 | <0.01 | 0.38 |
|  | **Baseline** | **48-weeks** | **Baseline** | **48-weeks** | **Baseline** | **48-weeks** |  |  |  |
| Section A, time, seconds | 6.2 (6.3) | 7.0 (5.6) | 4.5 (3.7) | 8.0 (7.2) | 5.6 (4.8) | 8.9 (6.9) | 0.17 | 0.51 | 0.14 |
| Section A, time, scaled | 5.9 (0.9) | 5.8 (0.7) | 5.9 (0.3) | 5.6 (0.8) | 5.8 (0.5) | 5.5 (1.0) | 0.27 | 0.37 | 0.54 |
| Section B, time, seconds | 38.2 (25.9) | 30.7 (25.0) | 42.7 (23.5) | 23.2 (13.6) | 27.0 (17.4) | 29.3 (16.7) | 0.69 | 0.18 | 0.17 |
| Section B, time, scaled | 5.5 (1.0) | 5.7 (0.7) | 5.4 (1.0) | 5.9 (0.4) | 5.7 (0.6) | 5.9 (0.4) | 0.56 | 0.98 | 0.26 |
| Category A errors, total | 0.6 (0.9) | 0.3 (0.6) | 0.9 (1.5) | 0.4 (0.7) | 0.7 (0.8) | 0.7 (0.9) | 0.14 | 0.11 | 0.88 |
| Category A errors, scaled | 1.8 (2.9) | 1.0 (1.7) | 2.9 (5.5) | 1.1 (2.4) | 2.2 (2.6) | 2.1 (3.0) | 0.13 | 0.10 | 0.91 |
| Category B errors, total | 2.0 (1.7) | 1.3 (1.1) | 2.0 (1.5) | 1.2 (1.4) | 1.4 (2.2) | 1.4 (1.9) | 0.56 | 0.59 | 0.84 |
| Category B errors, scaled | 2.7 (3.7) | 1.3 (1.1) | 2.3 (2.9) | 1.2 (1.4) | 2.6 (6.6) | 2.1 (5.0) | 0.21 | 0.24 | 0.67 |
| Total errors, scaled | 6.3 (1.2) | 7.0 (0.7) | 6.4 (1.4) | 7.1 (0.9) | 6.5 (1.8) | 6.7 (1.4) | 0.22 | 0.14 | 0.89 |
| Total scaled score | 17.8 (2.2) | 18.4 (1.3) | 17.6 (1.8) | 18.7 (1.6) | 18.0 (2.0) | 18.0 (1.9) | 0.19 | 0.11 | 0.84 |
| Overall scaled score | 5.8 (1.0) | 6.2 (0.6) | 5.8 (0.8) | 6.2 (0.8) | 6.0 (0.9) | 6.0 (0.8) | 0.40 | 0.38 | 0.90 |

Values are unadjusted means (SD). P1 = p-value for group using ANCOVA (adjusted for baseline value, study site, baseline age, and years of education), *= false discovery rate adjusted p value <0.05; P2 = p-value for contrast 1: Control v. (MD & MD+PA), P3 = p-value for contrast 2: MD v. MD+PA. MD = Mediterranean diet, PA = Physical activity. Data at 48-weeks were available for n=24 (MD+PA), n=22 (MD) and n=22 (Control).

**Table S10: Cognitive and cardiometabolic outcomes at baseline and 24-weeks by tertile of 24-week change in MEDAS score in 93 MedEx-UK participants.**

|  | **Tertile 1 (n=33)** | | **Tertile 2 (n=27)** | | **Tertile 3 (n=33)** | | **P=** | **FDR=** |
| --- | --- | --- | --- | --- | --- | --- | --- | --- |
|  | **Baseline** | **24-weeks** | **Baseline** | **24-weeks** | **Baseline** | **24-weeks** |  |  |
| General cognition | -0.03 (0.55) | -0.09 (0.64) | 0.01 (0.49) | -0.01 (0.55) | 0.10 (0.47) | 0.28 (0.52) | <0.01 | 0.04 |
| Processing speed | 0.03 (0.85) | -0.12 (1.1) | 0.02 (0.73) | -0.10 (0.85) | 0.20 (0.80) | 0.21 (0.83) | 0.18 | 0.26 |
| Executive function | -0.03 (0.51) | 0.09 (0.56) | -0.06 (0.67) | 0.05 (0.75) | 0.13 (0.59) | 0.41 (0.60) | 0.06 | 0.12 |
| Memory | 0.06 (0.75) | -0.04 (0.82) | -0.04 (0.78) | -0.02 (0.74) | -0.03 (0.68) | 0.22 (0.64) | <0.01 | 0.04 |
| Hayling: Section A, time, seconds | 5.2 (4.4) | 12.4 (8.3) | 5.7 (5.3) | 11.4 (5.7) | 5.9 (7.0) | 9.5 (7.5) | 0.03 | 0.10 |
| Hayling: Section A, time, scaled | 5.8 (0.57) | 5.2 (1.0) | 5.9 (0.66) | 5.3 (0.78) | 5.8 (0.81) | 5.4 (0.97) | 0.29 | 0.37 |
| Hayling: Section B, time, seconds | 30.3 (22.0) | 37.6 (29.1) | 40.2 (28.8) | 30.9 (13.3) | 32.4 (21.6) | 31.4 (22.1) | 0.15 | 0.23 |
| Hayling: Section B, time, scaled | 5.8 (0.78) | 5.5 (1.1) | 5.2 (1.1) | 5.9 (0.38) | 5.8 (0.78) | 5.8 (0.61) | 0.13 | 0.23 |
| Hayling: Category A errors, total | 0.70 (0.92) | 0.58 (0.71) | 0.59 (1.3) | 0.44 (0.64) | 0.67 (0.82) | 0.33 (0.54) | 0.14 | 0.23 |
| Hayling: Category A errors, scaled | 2.2 (3.0) | 1.7 (2.1) | 2.0 (4.9) | 1.3 (1.9) | 2.0 (2.5) | 1.0 (1.6) | 0.14 | 0.23 |
| Hayling: Category B errors, total | 1.6 (2.1) | 1.8 (2.1) | 1.9 (1.8) | 1.4 (1.7) | 1.9 (1.5) | 1.1 (1.1) | 0.04 | 0.10 |
| Hayling: Category B errors, scaled | 2.8 (5.9) | 2.9 (4.7) | 2.4 (3.6) | 1.8 (3.1) | 2.2 (2.7) | 1.1 (1.1) | 0.04 | 0.10 |
| Hayling: Total errors, scaled | 6.3 (1.8) | 6.3 (1.6) | 6.4 (1.5) | 6.8 (1.3) | 6.5 (1.1) | 7.0 (0.77) | 0.03 | 0.09 |
| Hayling: Total scaled score | 18.0 (2.2) | 16.9 (2.4) | 17.5 (2.3) | 18.0 (1.8) | 18.2 (1.5) | 18.2 (1.4) | <0.01 | 0.04 |
| Hayling: Overall scaled score | 6.0 (1.0) | 5.5 (0.94) | 5.7 (1.1) | 6.0 (0.81) | 6.1 (0.66) | 6.1 (0.63) | <0.01 | 0.03 |
| BMI, kg/m2 | 29.2 (4.4) | 29.2 (4.5) | 28.7 (4.2) | 28.3 (4.3) | 28.7 (4.9) | 28.4 (5.1) | 0.24 | 0.33 |
| 24hr mean SBP, mm Hg | 134.1 (8.8) | 133.5 (11.4) | 129.8 (15.5) | 128.5 (15.8) | 125.1 (11.7) | 123.6 (11.7) | 0.58 | 0.66 |
| 24hr mean DBP, mm Hg | 77.3 (7.4) | 76.4 (4.3) | 82.5 (12.8) | 76.2 (10.2) | 74.7 (6.2) | 75.3 (5.5) | 0.73 | 0.80 |
| 24hr mean PP, mm Hg | 56.7 (6.4) | 57.2 (9.3) | 52.1 (8.0) | 52.3 (8.4) | 50.4 (10.5) | 49.8 (9.5) | 0.45 | 0.54 |
| 24h SBP CV, mm Hg | 9.6 (1.5) | 9.5 (2.1) | 9.2 (1.8) | 9.2 (1.5) | 10.4 (2.7) | 9.8 (1.7) | 0.88 | 0.88 |
| 24h DBP CV, mm Hg | 10.5 (2.8) | 10.6 (2.7) | 8.6 (2.6) | 8.5 (2.4) | 10.7 (2.9) | 11.0 (3.0) | 0.80 | 0.83 |
| 24h PP CV, mm Hg | 14.1 (6.9) | 16.2 (8.6) | 11.2 (8.4) | 11.0 (8.1) | 16.4 (8.4) | 14.9 (8.0) | 0.01 | 0.06 |
| AASI | 0.58 (0.21) | 0.68 (0.12) | 0.59 (0.14) | 0.61 (0.16) | 0.58 (0.14) | 0.51 (0.13) | <0.01 | 0.03 |

Values are unadjusted means (SD). P = p-trend calculated using ANCOVA (adjusted for baseline value, study site, baseline age, and years of education); FDR= False discovery rate adjusted pvalue. Individual test scores were converted to Z scores standardised on Baseline grand mean and standard deviation with response time variables reversed by [Z * -1], so a higher time indicates a better outcome. Individual Z scores test scores were mean aggregated into Summary Scores for: Processing speed [Digit symbol substitution (total correct); Trail Making Test (A, seconds)]; Executive Function [Controlled Oral Word Association Test (total); Categorical verbal fluency test (total); Trail Making Test (B-A, seconds); Wechsler Memory Digit Span (backwards, total)] and Memory [Verbal paired immediate (total); Visual paired immediate (total); Verbal paired delayed (total); Visual paired delayed (total); Rey Auditory Verbal Learning Test (immediate); Rey Auditory Verbal Learning Test (recall)]. A general cognition score was calculated using all Processing speed, Executive function and Memory tests. Change in MEDAS score was: T1= 0.1 (SD 1.2) points; T2= 2.9 (SD 0.9) points; T3= 6.3 (SD 1.3) points. AASI = Ambulatory stiffness index, CV = variability, DBP = diastolic blood pressure, MD = Mediterranean diet, PA = physical activity, PP = pulse pressure, SBP = systolic blood pressure.

**Table S11: Cognitive and cardiometabolic outcomes at baseline and 24-weeks by tertile of 24-week change in moderate activity in 88 MedEx-UK participants.**

|  | **Tertile 1 (n=33)** | | **Tertile 2 (n=27)** | | **Tertile 3 (n=33)** | | **P=** | **FDR=** |
| --- | --- | --- | --- | --- | --- | --- | --- | --- |
|  | **Baseline** | **24-weeks** | **Baseline** | **24-weeks** | **Baseline** | **24-weeks** |  |  |
| General cognition | 0.09 (0.46) | 0.15 (0.56) | 0.09 (0.42) | 0.06 (0.53) | -0.17 (0.55) | -0.13 (0.64) | 0.78 | 0.86 |
| Processing speed | -0.07 (0.94) | -0.01 (1.0) | 0.16 (0.67) | 0.03 (0.99) | 0.10 (0.63) | -0.15 (0.84) | 0.10 | 0.44 |
| Executive function | 0.11 (0.53) | 0.21 (0.51) | 0.10 (0.57) | 0.14 (0.51) | -0.17 (0.68) | 0.22 (0.90) | 0.07 | 0.42 |
| Memory | -0.06 (0.66) | 0.08 (0.64) | 0.23 (0.48) | 0.20 (0.66) | -0.20 (0.91) | -0.21 (0.87) | 0.19 | 0.62 |
| Hayling: Section A, time, seconds | 5.8 (5.1) | 11.2 (7.5) | 5.2 (6.2) | 11.9 (6.9) | 5.6 (4.7) | 11.1 (8.2) | 0.99 | 0.99 |
| Hayling: Section A, time, scaled | 5.9 (0.51) | 5.2 (0.97) | 5.8 (0.73) | 5.2 (0.91) | 5.9 (0.62) | 5.3 (1.00) | 0.72 | 0.83 |
| Hayling: Section B, time, seconds | 33.0 (24.4) | 27.9 (15.5) | 32.6 (21.0) | 39.0 (23.8) | 35.6 (25.7) | 33.3 (28.0) | 0.51 | 0.79 |
| Hayling: Section B, time, scaled | 5.7 (0.98) | 5.9 (0.51) | 5.6 (0.82) | 5.7 (0.76) | 5.5 (0.95) | 5.6 (1.1) | 0.32 | 0.70 |
| Hayling: Category A errors, total | 0.67 (1.3) | 0.50 (0.63) | 0.72 (0.80) | 0.45 (0.57) | 0.62 (0.86) | 0.45 (0.74) | 0.67 | 0.83 |
| Hayling: Category A errors, scaled | 2.2 (4.8) | 1.5 (1.9) | 2.2 (2.5) | 1.3 (1.7) | 1.9 (2.8) | 1.3 (2.2) | 0.71 | 0.83 |
| Hayling: Category B errors, total | 1.8 (1.6) | 1.8 (2.1) | 1.9 (2.1) | 1.4 (1.4) | 1.7 (1.8) | 1.1 (1.3) | 0.05 | 0.42 |
| Hayling: Category B errors, scaled | 2.2 (2.9) | 2.8 (4.5) | 3.1 (6.1) | 1.7 (2.6) | 2.6 (4.1) | 1.2 (1.9) | 0.02 | 0.42 |
| Hayling: Total errors, scaled | 6.5 (1.5) | 6.5 (1.4) | 6.3 (1.5) | 6.7 (0.96) | 6.4 (1.4) | 6.9 (1.1) | 0.07 | 0.42 |
| Hayling: Total scaled score | 18.1 (2.2) | 17.6 (1.9) | 17.7 (1.8) | 17.7 (1.9) | 17.8 (1.9) | 17.7 (2.2) | 0.34 | 0.70 |
| Hayling: Overall scaled score | 6.0 (1.1) | 5.7 (0.74) | 5.9 (0.74) | 5.8 (0.82) | 5.9 (0.83) | 5.9 (0.94) | 0.18 | 0.62 |
| BMI, kg/m2 | 29.1 (4.3) | 28.8 (4.2) | 29.8 (4.3) | 29.7 (4.6) | 27.7 (4.7) | 27.4 (4.7) | 0.93 | 0.98 |
| 24hr mean SBP, mm Hg | 131.2 (13.2) | 132.0 (15.5) | 125.1 (15.3) | 123.0 (12.2) | 130.4 (10.3) | 127.2 (13.0) | 0.22 | 0.62 |
| 24hr mean DBP, mm Hg | 77.4 (9.6) | 76.4 (9.2) | 75.6 (11.2) | 72.1 (6.5) | 77.7 (7.4) | 77.9 (4.8) | 0.63 | 0.83 |
| 24hr mean PP, mm Hg | 55.4 (6.7) | 55.6 (9.2) | 51.5 (10.2) | 50.9 (7.9) | 52.7 (10.7) | 51.6 (11.3) | 0.44 | 0.79 |
| 24h SBP CV, mm Hg | 10.0 (2.0) | 9.8 (1.7) | 10.5 (2.4) | 9.6 (1.9) | 9.8 (2.3) | 9.5 (2.0) | 0.29 | 0.70 |
| 24h DBP CV, mm Hg | 10.9 (2.6) | 10.1 (2.9) | 10.2 (3.3) | 10.7 (3.4) | 9.7 (2.7) | 10.5 (2.8) | 0.39 | 0.75 |
| 24h PP CV, mm Hg | 14.8 (8.7) | 14.8 (8.8) | 16.3 (9.7) | 13.7 (7.4) | 15.3 (8.2) | 16.8 (9.4) | 0.64 | 0.83 |
| AASI | 0.67 (0.16) | 0.65 (0.13) | 0.62 (0.21) | 0.56 (0.14) | 0.55 (0.18) | 0.57 (0.18) | 0.50 | 0.79 |

Values are unadjusted means (SD). P = p-trend calculated using ANCOVA (adjusted for baseline value, study site, baseline age, and years of education); FDR= False discovery rate adjusted pvalue. Individual test scores were converted to Z scores standardised on Baseline grand mean and standard deviation with response time variables reversed by [Z * -1], so a higher time indicates a better outcome. Individual Z scores test scores were mean aggregated into Summary Scores for: Processing speed [Digit symbol substitution (total correct); Trail Making Test (A, seconds)]; Executive Function [Controlled Oral Word Association Test (total); Categorical verbal fluency test (total); Trail Making Test (B-A, seconds); Wechsler Memory Digit Span (backwards, total)] and Memory [Verbal paired immediate (total); Visual paired immediate (total); Verbal paired delayed (total); Visual paired delayed (total); Rey Auditory Verbal Learning Test (immediate); Rey Auditory Verbal Learning Test (recall)]. A general cognition score was calculated using all Processing speed, Executive function and Memory tests. Change in MEDAS score was: T1= -218 (SD 170) minutes; T2= 32.6 (SD 36.9) minutes; T3= 228 (SD 91.6) minutes. AASI = Ambulatory stiffness index, CV = variability, DBP = diastolic blood pressure, MD = Mediterranean diet, PA = physical activity, PP = pulse pressure, SBP = systolic blood pressure.

**Table S12: Planned behaviour change at baseline and 24-week by intervention group in 95 MedEx-UK participants.**

|  | **Baseline** | | | **Week 24** | | |
| --- | --- | --- | --- | --- | --- | --- |
|  | **MD + PA (n=32)** | **MD (n=31)** | **Control (n=32)** | **MD + PA (n=32)** | **MD (n=31)** | **Control (n=32)** |
| Perceived control to change diet | 4.4 (0.5) | 4.1 (0.8) | 4.2 (1.0) | 3.9 (0.8) | 4.0 (0.8) | 3.7 (0.8) |
| It would be difficult for me to change my diet, even if I wanted to | 4.3 (0.7) | 4.0 (0.9) | 4.3 (1.1) | 4.0 (0.9) | 3.9 (0.9) | 3.7 (1.0) |
| I am confident that I could change my diet, if I wanted to | 4.4 (0.6) | 3.9 (1.1) | 4.1 (0.9) | 3.9 (0.9) | 3.6 (1.1) | 3.8 (0.7) |
| Perceived control to increase PA | 4.4 (0.6) | 4.0 (0.9) | 4.1 (0.8) | 3.8 (0.8) | 3.6 (1.0) | 3.7 (0.6) |
| It would be difficult for me to become more physically active, even if I wanted to | 4.5 (0.7) | 4.1 (1.2) | 4.2 (1.2) | 3.8 (1.1) | 4.0 (0.8) | 3.6 (1.0) |
| I am confident that I could become more physically active, if I wanted to | 4.3 (0.7) | 4.1 (1.0) | 4.2 (1.0) | 3.8 (1.0) | 3.6 (1.1) | 3.7 (0.9) |
| Intention to change diet | 4.1 (0.7) | 3.9 (0.7) | 4.0 (1.0) | 3.2 (1.1) | 3.6 (1.2) | 3.2 (0.7) |
| It is likely that I will change my diet | 4.1 (0.7) | 4.0 (0.7) | 4.0 (1.0) | 3.3 (1.1) | 3.5 (1.2) | 3.1 (0.7) |
| I intend to change my diet | 4.0 (0.7) | 3.9 (0.9) | 4.0 (1.0) | 3.5 (0.9) | 3.5 (1.0) | 3.3 (0.7) |
| Intention to increase PA | 4.1 (0.7) | 3.9 (0.8) | 4.0 (0.8) | 3.6 (0.8) | 3.6 (1.0) | 3.4 (0.7) |
| It is likely that I will become more physically active | 4.1 (0.8) | 3.8 (0.8) | 4.0 (1.0) | 3.2 (1.2) | 3.7 (1.2) | 3.2 (0.9) |
| I intend to become more physically active | 4.2 (0.8) | 3.9 (0.8) | 4.1 (0.8) | 3.8 (0.8) | 3.7 (1.0) | 3.4 (0.8) |

Values are mean (SD) responses on a Likert scale from one to five, where higher numbers indicate a more positive response. MD = Mediterranean diet, PA = Physical activity.

**Table S13: Self-reported use of behaviour change techniques at 24-weeks in 69 MedEx-UK participants.**

|  | **MD + PA (n=24)** | **MD (n=23)** | **Control (n=22)** | **P-value** |
| --- | --- | --- | --- | --- |
| In the last six months…. |  |  |  |  |
| I set myself goals to eat a healthy diet | 4.6 (0.7) | 4.3 (0.7) | 3.4 (1.0) | <0.01 |
| I set myself goals to become more physically active | 4.2 (0.9) | 3.2 (1.1) | 3.4 (0.9) | <0.01 |
| I made detailed plans for how to eat a healthy diet | 3.9 (1.2) | 3.5 (1.0) | 2.2 (1.1) | <0.01 |
| I made detailed plans for how to become more physically active | 3.8 (1.0) | 2.6 (1.2) | 2.3 (1.1) | <0.01 |
| I kept track of my diet | 4.2 (0.9) | 4.0 (0.9) | 2.7 (1.1) | <0.01 |
| I kept track of my physical activity levels | 4.1 (1.0) | 2.9 (1.2) | 2.7 (1.3) | <0.01 |
| I reviewed progress with my goals to eat a healthy diet | 3.9 (1.0) | 3.7 (0.9) | 2.3 (1.1) | <0.01 |
| I reviewed progress with my goals to become more physically active | 3.9 (0.9) | 2.5 (1.0) | 2.3 (1.2) | <0.01 |
| I asked others to support me with eating a healthy diet | 3.2 (1.5) | 3.3 (1.2) | 2.3 (1.4) | 0.01 |
| I asked others to support me with becoming more physically active | 2.9 (1.2) | 2.4 (1.2) | 2.4 (1.3) | 0.12 |
| I looked for solutions when I experienced problems in eating a healthy diet | 3.4 (0.9) | 3.5 (0.9) | 2.6 (1.4) | <0.01 |
| I looked for solutions when I experienced problems in becoming more physically active | 3.5 (0.9) | 2.7 (1.1) | 2.5 (1.4) | <0.01 |
| I rewarded myself when I achieved my goals in eating a healthy diet | 2.0 (1.1) | 2.6 (1.0) | 2.0 (1.3) | 0.89 |
| I rewarded myself when I achieved my goals in becoming more physically active | 2.0 (1.1) | 2.0 (0.9) | 1.9 (1.1) | 0.82 |
| I made eating a healthy diet part of my daily routine | 4.5 (0.6) | 4.3 (0.9) | 3.4 (1.2) | <0.01 |
| I made physical activity part of my daily routine | 3.9 (1.0) | 2.9 (1.2) | 3.2 (1.2) | 0.03 |

Values are mean (SD) responses on a Likert scale from one to five, where higher numbers indicate a more positive response. P-value for group using ANCOVA (dependent variable= value at 24 weeks; independent variables= treatment group; covariates= study site). Missing data; ^1^ n=1. MD = Mediterranean diet, PA = Physical activity.
